# Characterizing the Stoichiometry of Individual Metal Sulfide and Phosphate Colloids in Soils, Sediments, and Industrial Processes by Inductively Coupled Plasma Time-of-Flight Mass Spectrometry

**DOI:** 10.1021/acs.est.3c10186

**Published:** 2024-06-25

**Authors:** Jonas Wielinski, Xiaopeng Huang, Gregory V. Lowry

**Affiliations:** Department of Civil and Environmental Engineering, Carnegie Mellon University, Pittsburgh, Pennsylvania 15213, United States

**Keywords:** colloids, nanoparticles, particle, sulfides, phosphates, metals, mass spectrometry

## Abstract

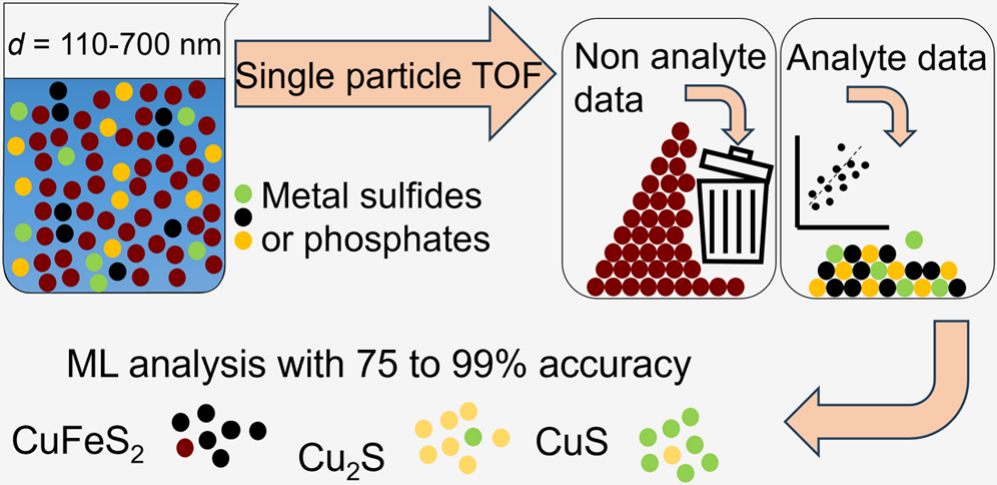

Size and purity of metal phosphate and metal sulfide colloids can control the solubility, persistence, and bioavailability of metals in environmental systems. Despite their importance, methods for detecting and characterizing the diversity in the elemental composition of these colloids in complex matrices are missing. Here, we develop a single-particle inductively coupled plasma time-of-flight mass spectrometry (sp-icpTOF-MS) approach to characterize the elemental compositions of individual metal phosphate and sulfide colloids extracted from complex matrices. The stoichiometry was accurately determined for particles of known composition with an equivalent spherical diameter of ≥∼200 nm. Assisted by machine learning (ML), the new method could distinguish particles of the copper sulfides covellite (CuS), chalcocite (Cu_2_S), and chalcopyrite particles (CuFeS_2_) with 75% (for Cu_2_S) to 99% (for CuFeS_2_) accuracy. Application of the sp-icpTOF-MS method to particles recovered from natural samples revealed that iron sulfide (FeS) particles in lake sediment contained ∼4% copper and zinc impurities, whereas pure pyrite (FeS_2_) was identified in hydraulic fracturing wastewater and confirmed by selected area electron diffraction. Colloidal mercury in an offshore marine sediment was present as pure mercury sulfide (HgS), whereas geogenic HgS recovered from an industrial process contained ∼0.08 wt % silver per Hg, enabling source apportionment of these colloids using ML. X-ray absorption spectroscopy confirmed that Hg was predominantly present as metacinnabar (β-HgS) in the industrial process sample. The determination of impurities in individual colloids, such as zinc and copper in FeS, and silver in HgS may enable improved assessment of their origin, reactivity, and bioavailability potential.

## Abstract 2

Colloidal metal sulfides and metal phosphates are important phases in environmental systems that can be difficult to detect. Our new method allows for the rapid characterization of these colloids in environmental and technical systems.

## 1 Introduction

Metal phosphates and sulfides occur as colloids in many natural, anthropogenically impacted, and engineered systems. For example, metal sulfides are found in ore deposits, marine and freshwater sediments,^1,2^ soils,^3^ chemical process chains,^4^ or engineered wastewater treatment systems^5,6^ such as constructed wetlands.^7^ Iron sulfide(s) are more reactive compared to some iron (oxy)hydroxides and play an important role in biogeochemical Fe and S cycles and in contaminant mobilization,^4,9,10^ especially if they or other metal sulfides occur as nanoparticles (NPs).^5,8−14^ Iron sulfides such as pyrite can also be released following anthropogenic activity, e.g., in mine tailing or unconventional natural gas extraction (hydraulic fracturing), where they can pose a threat to the natural environment.^15,16^ In lead-contaminated soils, phosphate can be added to induce the precipitation of the colloidal lead (Pb) phosphate pyromorphite (Pym).^17−22^ Pym precipitation significantly reduces the Pb bioavailability and thus enables repurposing contaminated sites. Similarly, the ultrafine particulate fraction in air (particulate matter smaller 2.5 μm, referred to as PM2.5) also contains metal phosphates^23^ and sulfides,^24^ which can exhibit adverse effects toward humans.^25^ In general, colloids (and nanoscale precipitates) have shown to exhibit greater reactivity compared to flat cleaved surfaces.^26^ Thus, this size fraction is important for assessing metal toxicity toward aquatic wildlife^27^ and humans.^28^

A considerable share of the available literature relies on electron microscopy (EM) and X-ray absorption spectroscopy (XAS) to characterize metal phosphate or sulfide colloids in complex environmental matrices. EM and XAS produce different kinds of particle characterization information with a high level of certainty but also suffer some drawbacks. For instance, XAS requires in-depth knowledge about the sample, is not suitable for rapid screening of a large number of samples, provides only an “average” metal speciation rather than information on individual particles, and requires element concentrations in samples to be of at least tens to hundreds of parts per million (ppm). EM, on the other hand, provides particle specific information, but requires a high analyte particle number concentration in the sample to find them, (i.e., a relatively high analyte concentration) and a large number of measurements are needed to obtain good particle counting statistics.^29^

Analytical techniques that can rapidly characterize thousands of individual (metal) phosphate and sulfide colloids present in complex matrices are needed to elucidate their fate and reactivity in environmental, biological, and relevant industrial matrices. Metal (nano) particles can be detected by single-particle inductively coupled plasma time-of-flight mass spectrometry (sp-icpTOF-MS), which can rapidly and easily measure tens-of-thousands of particles made from elements spanning a large proportion of the periodic table (from magnesium to uranium) in the size range from around 30 nm to several hundreds of nanometers.^30−33^ Thus, with modern computational capabilities, detecting a small number of target particles in a much greater number of nontarget particles becomes feasible, even at a low analyte concentration. For example, to detect a small share of iron sulfide in a sediment dominated by goethite (α-FeOOH) by XAS, a longer measurement time will improve the signal-to-noise ratio, but the measured proportion of α-FeOOH to iron sulfide will remain constant, and the less concentrated sulfide phase will eventually not be discernible. In contrast, if sediment colloids are measured individually by sp-icpTOF-MS, nontarget particles such as α-FeOOH or any other metal sulfide can simply be ignored, while target FeS particles are kept for evaluation. Thus, in contrast to XAS, for sp-icpTOF-MS, the target colloidal signal is independent of the nontarget colloidal signals, assuming that particle number concentrations do not exceed a threshold leading to two or more particles arriving at the detector at the same time.

Both sulfur and phosphorus are important metal ligands in many environmental and biological matrices. However, relatively high S and P ionization energies (10.4 and 10.5 eV for S → S^+^ and P → P^+^, respectively) lead to low ionization efficiencies of around 14% and 30% for S and P, which are much lower compared to those of many metals that are close to 100%.^34,35^ Unfavorable mass bias at the relatively low masses further reduces the measured sensitivities.^34^ Two recent studies laid out the capabilities of different icp-MS to detect P in individual cells with or without Ag-NPs, but details of the P detection were not reported.^33,36^ Multiple studies have reported detection of individual sulfide containing NP by sp-icp(TOF)-MS.^37−40^ However, due to polyatomic interferences at the main isotope of S (^32^S), the available literature only reports detecting the metal cation(s) in particles and assumes the balance as S for the calculation of particle diameters. The ability to measure both the metal and P and S in particles simultaneously will enable the determination of particle stoichiometry and purity to infer information about origin, reactivity, and fate. Therefore, in this study, we provide a rapid and easy workflow to detect metals and the corresponding P or S in colloids recovered from complex environmental matrices by sp-icpTOF-MS in the oxygen reaction mode.^41^ The developed method was successfully tested and validated using different synthetic colloids and milled natural minerals of known stoichiometry and then used to measure metal sulfides in a hydraulic fracturing wastewater, an urban lake sediment, an off-shore marine sediment, an industrial process stream, and Pb phosphate colloids in a Pb-contaminated soil before and after P treatment. Measurements of metal sulfides and phosphates in real matrices showed differing levels of impurities at the particle level of relevance to particle stability, toxicity, and the determination of origin.

## 2 Materials and Methods

### 2.1 Sp-icpTOF-MS Operation in Oxygen Reaction Mode and Particle Detection

Phosphorus and S are challenging to quantify by icp-MS due to, e.g., polyatomic interferences, such as ^15^N^16^O^+^ or ^30^Si^1^H^+^ for ^31^P and ^16^O_2_^+^ or ^14^N^18^O^+^ for ^32^S.^42,43^ Therefore, P quantification utilizes either hydrogen (H_2_) or O_2_ as reaction gas to form, ^31^P^1^H^+^-^31^P^1^H_4_^+^ or ^31^P^16^O^+^ [mass/charge (*m*/*z*) 47], respectively. For S quantification, O_2_ is used as a reaction gas to form ^32^S^16^O^+^ (*m*/*z* 48). However, both *m*/*z* 47 and 48 suffer from other interferences present in natural samples, e.g., ^47^Ti^+^, ^48^Ca^+^, and ^48^Ti^+^. On an ICP triple quadrupole (QQQ) MS, with two mass separators upstream and downstream of a reaction cell, these interferences can be eliminated, whereas an icpTOF possesses no such option and the resolving power of ∼3000 of the used instrument (icpTOF R, TOFWERK AG, Thun, Switzerland) was insufficient to separate between the analyte and a polyatomic interference. Therefore, in this study, we reacted ^31^P and ^32^S with ^16^O and the following sample and isotope specific inclusion rules were set to minimize mislabeling particle events: for a particle to contain P, *m*/*z* 47 must be detected but neither *m*/*z* 46 nor *m*/*z* 48; for a particle to contain S, *m*/*z* 48 must be detected but neither *m*/*z* 46 nor *m*/*z* 47 (Table S1).

These rules addressed interferences resulting from Ti and Ca. Besides detection rules for S and P, we showed that accounting for multiple isotopes per element improved the agreement between dynamic light scattering (DLS) and TOF-based particle size distributions and was thus applied in this study to reduce the number of false positive detects (Table S1 and Supporting Information, Section S1).

An icpTOF R was operated either in the O_2_ reaction mode or without the addition of a reaction gas (here called “no-gas mode”). The icpTOF R is a hybrid instrument with an iCap RQ (Thermo Fisher) front-end with the quadrupole mass separator exchanged for a notch filter that can be used to attenuate four *m*/*z* ratios with variable strengths and a TOF mass separator (TOFWERK AG, Thun, Switzerland).^31^ In O_2_ reaction mode, the O_2_ flow rate into the iCap RQ collision cell was set to 0.2 mL/min. Read values fluctuated between 0.13 and 0.17 mL/min. This injection rate was a compromise between relative O_2_ mass flow fluctuation and ^31^P^16^O^+^ sensitivity at *m*/*z* 47. The sensitivity remained constant at increasing P loads up to at least 2000 ppm and in the presence of a 20 ppb 71-element standard simulating a high-dissolved metal background (results not shown). The sample introduction was facilitated by a self-aspirating perfluoroalkoxy (PFA) nebulizer with a gravimetrically measured uptake rate of 51 μL/min (PFA-50 MicroFlow, Elemental Scientific) (similar to^44^) inserted into a 47 mm PFA linear spray chamber (Elemental Scientific) combined with a 1.8 mm sapphire injector (Elemental Scientific) set to 6 mm sampling depth. This sample introduction system showed improved performance compared to a standard sample introduction setup using a peristaltic pump (details in Supporting Information, Section S2). Nickel (Ni) cones were used. An identical configuration was used for S detection.

Before each run, the instrument was auto tuned to either ^31^P^16^O^+^ for measurements of P or ^32^S^16^O^+^ for S measurements using a 100 ppb P or S solution (representative instrument tune parameter values in Table S2). The collision cell flat pole quadrupole (CCT) bias, modulating the collision energy, was tuned manually to −2.0 V to optimize the P or S sensitivity. The sensitivities were around 116 ± 1 cps/ppb for P and 110 ± 3 cps/ppb for S and thus much lower compared with most sensitivities of relevant metal isotopes (Table S3). Every 3 runs with around 5 h of data acquisition per run, the instrument was brought to standard configuration (MicroMist glass nebulizer, cyclonic nebulization chamber, 2.5 mm injector, 5 mm sampling depth, no-gas mode, and 0.4 mL/min sample flow) and tuned using an iCap Q tune solution (1 ppb cobalt, indium, cerium and uranium in 2% nitric acid and 0.5% hydrochloric acid, Thermo Fischer) and the manufacturer’s standard tune setting. The microchannel plate detector was tuned in the no-gas mode to reach a single ion value of 2.5 in accordance with the manufacturer’s recommendation to ensure relatively constant sensitivities for comparison between instrument runs.

For some Pb measurements, one of the four notch filters was set to *m*/*z* 209 and 0.5 V. This suppressed the Pb signal, and larger Pb containing colloids fell within the linear range of the detector (see discussion in Supporting Information, Section S3). For the separation of particle events from the background signal, the liquid reprocessing software code (TOFWERK AG, Thun, Switzerland) was used. The background signal was assumed to be Poisson distributed, and a particle event was identified if a recorded signal exceeded the threshold defined in eq 1
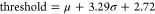
1with the average (μ) and standard deviation (σ) of signal intensity, respectively.^45^ Averaging windows of 100 measurement points was used, and a particle event was allowed to last for up to 3 dwell times. Up to 100 iterations were performed per averaging window, and particle masses were exported. All other computations were performed by using Matlab 2020b (MathWorks Inc.) or Python3. Details on external calibrations are available in Supporting Information, Section S4.

### 2.2 Colloid Synthesis and Characterization

Colloids of known phases were synthesized or obtained by milling to confirm that sp-icpTOF-MS could determine the correct stoichiometry of metals and phosphate and sulfide at the same time. Synthesized colloids were characterized by X-ray diffraction, transmission electron microscopy, and DLS. They included pyromorphite (referred to as Pym), chalcopyrite (CuFeS_2_), and sphalerite (ZnS). In addition, covellite [CuS (milled)], chalcocite [Cu_2_S (milled)], and pyrite [FeS_2_ (milled)] colloids were obtained by milling minerals of geogenic origin. Details on preparation and characterization are available in Supporting Information, Section S5.

### 2.3 Extraction of Colloids from Environmental Samples

We obtained a range of environmental samples containing metal-sulfide and metal-phosphate colloids. The samples included a hydraulic fracturing wastewater, a subaquatic lake sediment, an offshore marine sediment, a water/organic solvent mixture from an industrial scale process (referred to as NGP) and Pb-contaminated soil from a rifle range before and after treatment with phosphoric acid for Pym precipitation. Colloids were extracted following a modified protocol indicated in Bland et al.^46^ and measured by sp-icpTOF-MS. Briefly, in the environmental samples were suspended in 10 mM NaCl for cation exchange to disaggregate colloids in the presence of a mono valent electrolyte, washed three times with DI water by centrifugation, stabilized using carboxymethyl cellulose, treated with bath or probe sonication, and diluted in DI water. The protocol was optimized for the different sample matrices and varied slightly between samples. Details on the preparation of each sample are available in Supporting Information, Section S6. The evaluation of data collected on the Pb-contaminated soil before and after phosphoric acid treatment is described in Supporting Information, Section S7. The evaluation of all other samples is described below.

## 3 Results and Discussion

### 3.1 Detection of Metal Phosphate or Sulfide Stoichiometry in Colloids of Known Composition and Classification by Machine Learning

Pure phase synthetic Pym and ZnS with known stoichiometry were measured by sp-icpTOF-MS in the O_2_-reaction mode to determine if the metal and P or S can be measured simultaneously and return the correct stoichiometry. Results are reported in diameters (in nm), although particles were partially aggregated (Figure S1). Where aggregated pure-phase particle events were measured, the reported diameter indicated the diameter of a sphere that would have the same number of atoms as the agglomerate measured. However, since pure phase minerals with known composition were measured, this does not compromise the ability to confirm that the method returns a correct stoichiometry or to determine the approximate particle mass detection limits. In addition, using Cu_2_S, CuS (both milled), and CuFeS_2_, we demonstrated distinguishing between different Cu–S stoichiometries for a rudimentary speciation analysis potentially extrapolatable to other mineral phases.

**Figure 1:**
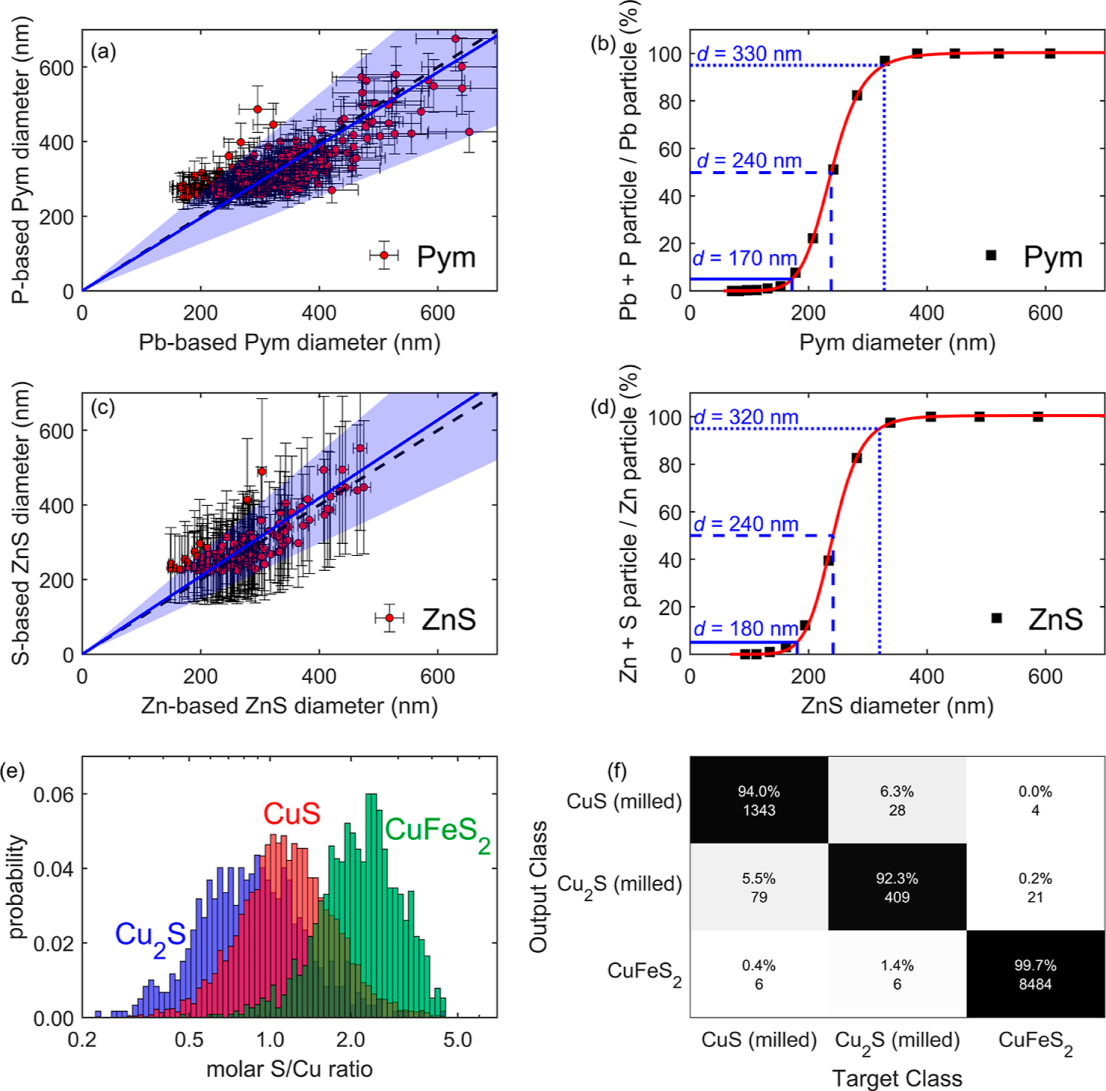
(a–d) Data recorded on synthesized Pym and ZnS colloids and (e–f) three copper sulfides of known composition were used to demonstrate the capabilities of ML for particle classification. (a) P-based diameters of Pym (nm) vs Pb-based diameters of Pym (nm). A measurement reflecting the ideal stoichiometry of Pym [Pb_5_(PO_4_)_3_Cl] falls on the dashed 1:1 line. Error bars in the *x* and *y* directions indicate two standard deviations obtained by propagating the uncertainty of the Pb and P calibration standards, respectively. Shaded areas above and below the solid blue regression lines indicate ±2σ of the regression. (b) True positive P detection as a function of Pym dimeter (nm). The red curve indicates a logistic model fit. The horizontal and vertical blue lines indicate the sizes at which in 5, 50 and 95% of Pb particles where P was also detected. (c) Sample ZnS: S-based ZnS diameter vs Zn-based ZnS diameter. Only every fifth data point (20% of the data) is shown for clarity. The uncertainty interval was calculated on the entire data set. Error bars are propagated from uncertainty in Zn and S calibration standards. (d) True positive S detection as a function of particle size. The red curve indicates a logistic model fit. The horizontal and vertical blue lines indicate the sizes at which in 5, 50 and 95% of Zn particles where S was also detected. (e) Ratios of S/Cu for Cu_2_S (milled), CuS (milled), and CuFeS_2_ from sp-icpTOF-MS data. (f) Confusion matrix for results obtained after training and testing on 75 and 25% of a data set containing Fe, Cu, and S masses, respectively.

We calculated the Pym-diameter in *n* = 251 particle events using either the Pb signal or the P signal recorded for that particle event, assuming the ideal stoichiometry of Pym (Pb_5_(PO_4_)_3_Cl), spherical geometry, and a density of ρ_Pym_ = 7.04 g/cm^3^. Plotting the calculated particle size based on Pb content versus the calculated particle size based on the P content (Figure 1a) will yield a straight line with a slope of 1 if both estimates return the same expected stoichiometry for Pym. The same size estimate based on either the measured Pb or the P in the particles confirms the particle’s phase. We find a good agreement between the ideal and calculated sizes for *d*_Pym_ > ∼240 nm (∼0.18 fmol P/particle; LOD calculated from eq 1 was 100 nm, Table S3). Below *d* ∼ 240 nm, the mass of P was overestimated, although this can be corrected for empirically using measurement results of colloids of the Pym-mimetite (Pym-Mim) solid solution (details in Supporting Information, Section S8 and Figure S2).

We plotted the frequency (%) of simultaneously detecting P in the Pb-bearing particles (denoted as a true positive) as a function of particle size (Figure 1b). This concept is applicable because all Pym particles should contain both Pb and P. The smallest particles may record only Pb because the LOD for Pb is lower than for P (Table S3). A logistic model of the form *a*/{1 + exp[−b · (*d*_Pym_ – *c*)]} with *a*, *b*, and *c* being fitting parameters described the resulting true positive detection rate as a function of particle size and provides a robust estimate of the LOD for P per particle. Phosphorus detection requirements from 5 to 95% of the particles led to particle LODs of *d* = 170 to 330 nm. Applying an orthogonal Poisson statistics-based approach using a false negative rate of 5% (i.e., losing 5% of the particles), we find a detection limit of *d* = 360 nm for the detection Pb and P in Pym,^47^ which is in good agreement with a 95% P detection requirement (330 nm) determined above.

ZnS particle events (*n* = 725, ρ_ZnS_ = 4.09 g/cm^3^) scattered around the 1 to 1 line with a good agreement for *d* = 200–500 nm (Figure 1c). At lower sizes around 200 nm, close to the S LOD (LOD from eq 1 was 100 nm, Table S3), the S concentrations were overestimated compared to Zn, likely due to a similar effect as observed above for P versus Pb in Pym. In analogy to the size-dependent percentage of Pb particles in which P was also detected, we show that the relative detection of Zn and S in ZnS followed the same trend (Figure 1d). A logistic model of the same form as indicated above-described the resulting true positive detection rate as a function of particle size and provides a robust estimate of the LOD for S per particle. Sulfur detection requirements from 5 to 95% of the particles led to particle LODs of *d* = 180 to 320 nm. The data for ZnS were obtained on a sample that was washed by centrifugation and with DI water several times, meaning that it had low Zn and S background concentrations leading to low particle LODs. Environmental samples will potentially have higher values for LODs, but lower values are unlikely. In conclusion, the presented method was capable of detecting ZnS of *d* > 110 nm returning accurate stoichiometry for *d* > ∼200 nm (∼0.16 fmol S/particle) and S was detected in 95% of the particles from *d* = 320 nm.

Copper sulfides can have varying Cu/S molar ratios (Figures 1e and S3) that can potentially be differentiated using the sp-icpTOF-MS method developed here. Classification using ML, more precisely supervised k nearest neighbor (KNN) clustering^48^ could distinguish between covellite (molar Cu/S ratio 1:1), chalcocite (Cu/S 2:1), and chalcopyrite (Cu/S 1:2) based on Cu, Fe, and S masses in individual particles without the need to convert them into sizes a priori (details in Section S9). The accuracy of predicting the correct phase of a particle event depended on the mineralogy and is shown in Figure 1f. As expected, the method could distinguish CuFeS_2_ with an accuracy of ≥99%, which resulted from the availability of an Fe signature distinguishing CuFeS_2_ from both CuS and Cu_2_S. In addition, the high S/Cu ratio of 2 in CuFeS_2_ distinguished these particles in the histogram (Figure 1e). In contrast, ∼19% (79/409 ≈ 19%, Figure 1e) of Cu_2_S particles were mislabeled as CuS particles. This was likely because the low ratio of S/Cu in Cu_2_S particles led to stronger nonlinearity effects for particles of the same size as compared to CuS or CuFeS_2_. Nonlinearity effects emerge because the sensitivity for Cu is much higher compared to S leading to detector saturation at lower sizes for higher ratios of Cu to S (Table S3 and Figure S3c,d). Thus, Cu_2_S appeared to be CuS. However, the absolute Cu and S masses were also an important distinguishing feature, not only their ratio. In Figure 1e, the overlapping fractions of CuS and Cu_2_S were not necessarily of the same size. For example, for Cu_2_S, the ratio of Cu/S decreases due to detector nonlinearity at a smaller mass of S, which is an additional feature that enables distinction between CuS and Cu_2_S.

Overall, the ML was capable of distinguishing between different copper sulfides, especially for those particles with multiple elements and high S to metal ratios. These analyses indicate an ability to discern particle stoichiometry based on individual particle measurements by sp-icpTOF-MS and can thus be used for a rudimentary speciation analysis. This method was applied to real particles recovered from a range of environmental matrices, as described in the sections below.

### 3.2 Pure Pyrite Colloids in Hydraulic Fracturing Wastewater and Impure Iron Sulfide Colloids in an Urban Freshwater Lake Sediment

**Figure 2:**
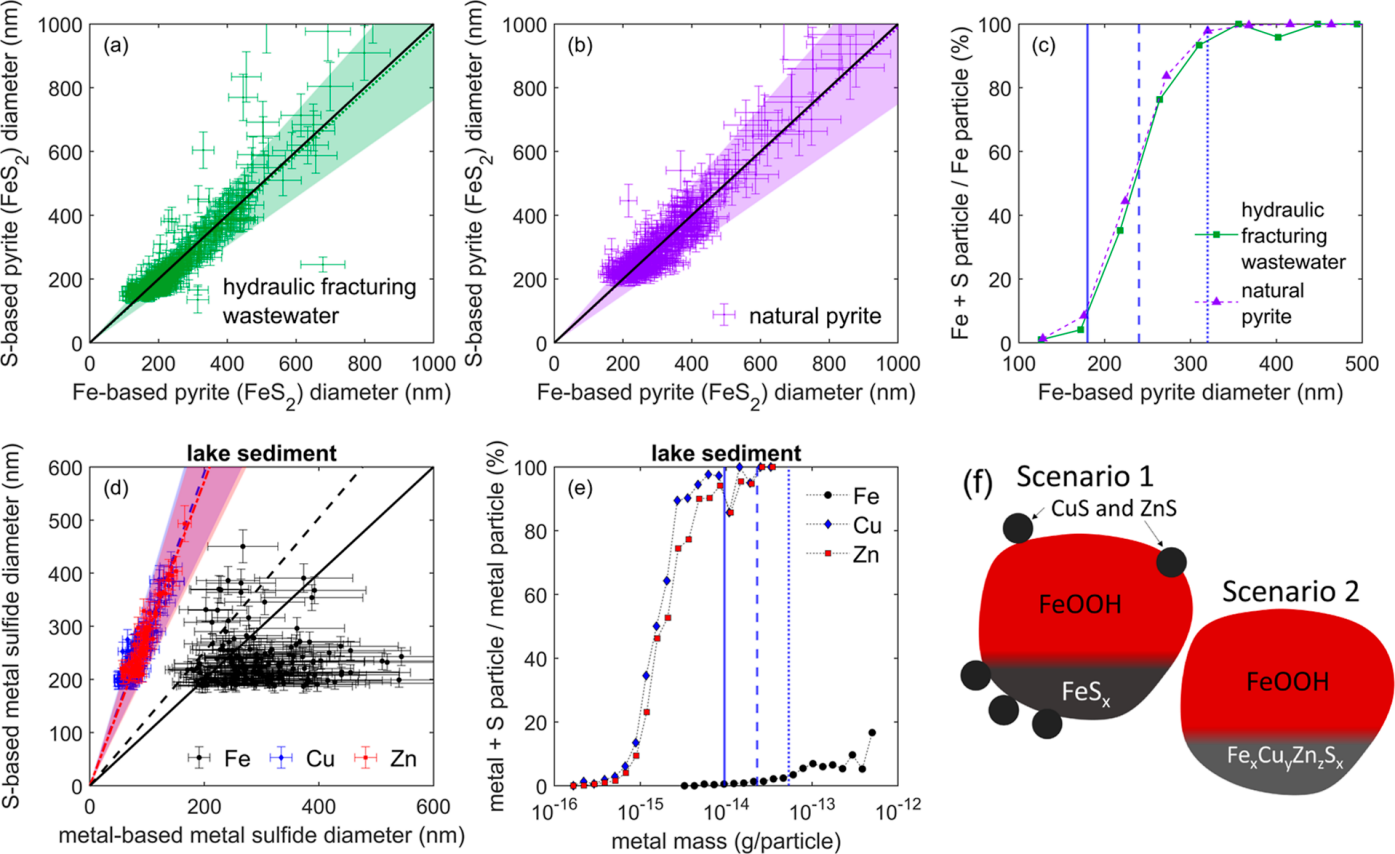
Metal sulfides in a hydraulic fracturing wastewater, natural pyrite for comparison, and urban freshwater lake sediment sample. Panels (a,b): scatter plots of the S-based pyrite (FeS_2_) diameter versus Fe-based pyrite diameter for particles in the hydraulic fracturing wastewater and for a milled, natural pyrite sample, respectively. Green or violet dots and error bars indicated sample data, the dotted, lines, and shaded area indicate a regression and ±2σ, respectively. The bold, black line indicates the ideal value for pyrite. (c) Relative frequencies of particles containing S and Fe divided by those that contain at least Fe (green squares and bold line: hydraulic fracturing wastewater, purple triangles and dashed line: natural pyrite) in percent. The vertical blue lines indicate the diameters (180, 240, and 320 nm) at which S should be detected in 5, 50, and 95% sulfide containing particles derived from ZnS data (Figure 1d). (d) S-based metal sulfide diameter vs metal-based metal sulfide diameter in particles collected on the lake sediment sample. The solid black line indicates a 1 to 1 ratio (e.g., FeS), and the dashed black line indicates a 0.5 to 1 ratio (e.g., FeS_2_). The dashed blue line indicates 3.9 mol % Cu replacing Fe in FeS and the dashed-dotted red line represents 4.3 mol % Zn replacing Fe in FeS. Both values represent fitting results, and the shaded areas indicate ±2σ. For Cu and Zn, only every second and every firth measurement value is shown, respectively, for clarity. (e) Relative frequencies of particles containing S in the lake sediment: Fe (black circles), Cu (blue diamonds), or Zn (red squares) divided by the frequencies of particles containing at least Fe, Cu, or Zn, respectively, as a function of mass. The vertical lines indicate metal masses at which 5, 50 and 95% of a metal-sulfide colloid should be detected based on data derived from synthesized ZnS. (f) Two possible scenarios for the occurrence of Cu and Zn with iron (sulfide). In scenario 1, CuS and ZnS occur as individual particles independent of FeS, whereas in scenario 2, Fe, Cu, Zn, and S occur as a single phase.

Naturally occurring FeS_2_ (*n* = 560) was detected in a hydraulic fracturing wastewater (Figures 2a and S4a). The molar ratio of S/Fe was 1.83 ± 0.29 (±2σ), close to the expected value of 2 and the data agreed well with the stoichiometry of a milled natural pyrite (Fe_2_S) sample (Figure 2b). At *d* ≥ 320 nm, all Fe was associated with S (Figure 2c). This size agrees well with the size of *d* = 320 nm at which S was detected in 95% of ZnS particles (vertical dotted 95%-line,Figure 2c). These results suggested that FeS_2_ was the quantitatively dominant Fe-phase in the hydraulic fracturing fluid. Selected area electron diffraction confirmed the presence of pyrite in hydraulic fracturing wastewater (Figure S5). In the Permian Basin, where the hydraulic fracturing took place, Fe is often present as pyrite, in agreement with our data.^15,49^ Earlier reports using ex situ laboratory experiments with shale samples containing high pyrite concentrations (3 wt %) reported that pyrite released dissolved Fe^2+^, which is then mobilized and oxidized into Fe^3+^ in hydraulic fracturing fluid.^50^ The findings of our study suggest that directly released colloidal pyrite may also be a relevant mobilized Fe-phase during hydraulic fracturing. Furthermore, the identification of pyrite confirmed that the sp-icpTOF-MS method is capable of correctly identifying metal sulfides in a complex matrix, thus inspiring confidence in data collected on more complex mineral phases.

The sp-icpTOF-MS analysis was also able to distinguish sulfide containing particles in urban freshwater lake sediment (*n* = 692 S containing particles). Most of these particles were associated with Zn, Cu, and Fe, as well as other elements (Figures 2d and S4b). The Fe-based calculated diameters are larger than the sulfur-based diameters and are poorly correlated, indicating a range of particle stoichiometries of Fe_*x*_S_*y*_, where *x* > *y*, i.e., Fe rich. In contrast, the sulfur-based diameters for CuS (ρ_CuS_ = 4.7 g/cm^3^) and ZnS are greater than the metal-based diameters, and Cu and Zn are highly correlated with sulfur. This indicates an excess of sulfur relative to Cu and Zn. The strong correlation of metal and S for chalcophile Cu and Zn indicates that these elements are fully sulfidized, accounting for ∼3.9 and ∼4.3% of the sulfur in their particles (Figure 2d). The lack of correlation between the Fe and sulfur signals renders the presence of a well-defined Fe-sulfide mineral such as shown for pyrite in hydraulic-fracturing wastewater unlikely. We hypothesize that the difference was rooted in the less complete sulfidation of Fe compared to Cu and Zn, the two latter being chalcophile elements, thus leaving room for higher Fe/S ratios in the particles.^51^ This is supported by the fact that the larger Zn and Cu particles were 100% associated with sulfur, whereas only 18% of the larger Fe containing particles also has sulfur (Figure 2e), which pointed toward the presence of sulfidized and nonsulfidized Fe-colloids.

Comparing the metal masses at which in 5–95% of ZnS particles S was detected in addition to Zn (calculated from ZnS dimeters in Figure 1d) to those of Cu and Zn extracted from the lake sediment, we find that the masses in the lake sediment are much lower (Figure 2e). This indicates that in particles containing Cu (and/or Zn) and S there must be another phase containing S. Otherwise, the relative detection frequencies should match, as shown for pyrite in the hydraulic fracturing wastewater (Figure 2c). In conclusion, most of the Fe–S particles in the lake sediment showed a higher Fe/S ratio, indicating that most Fe-colloids in the sediment were likely present as Fe (oxy)hydroxides of which a share was partially reacted with S to form Fe sulfides or to which Fe sulfide and Cu/Zn sulfide was attached.

These data also provided further insights into how Cu and Zn were affiliated with the FeS particles. Cu and Zn could be individual smaller particles of CuS or ZnS (or similar) attached to larger Fe-colloids or form an impure Cu_*x*_Zn_*y*_Fe_*z*_S phase (Figure 2f). Distinguishing between these two conceptual models is required to predict the correct dissolution behavior and bioavailability potential of Cu and Zn in the sediment.^52,53^ The Cu and Zn to S relationships measured in individual colloids pointed to the formation of a single phase. The attachment of smaller CuS and ZnS to larger FeS-containing colloids would lead to a loss of correlation between Cu, Zn, and S, because a random number of smaller CuS and ZnS could attach to the larger FeS and the FeS contributes the majority of the S signal in the particle (Figure 2f, scenario 1). However, Cu and Zn were well correlated to S at the individual particle level (and to each other; see Figure S6), indicating that Fe, Cu, Zn, S, and potentially other unidentified elements formed a single phase with diameter equivalents of 150–500 nm for a phase of the form Cu_0.039_Zn_0.043_Fe_0.923_S or similar rendering scenario 2 more likely (Figure 2f). Natural pyrrhotite (Fe_(1-*x*)_S; *x* ≤ 0.2) was shown to contain up to 0.4 wt % of Cu,^54^ which is lower than the 3.9% found in this study, whereas synthetic mackinawite has been prepared containing up to 20 mol % Cu (Cu_0.2_Fe_0.8_S) suggesting that a phase such as Cu_0.039_Zn_0.043_Fe_0.923_S could be stable in a lake sediment.^55^

The presence of a single Cu_0.039_Zn_0.043_Fe_0.923_S phase (or similar) could have consequences for the release of Cu and Zn under oxidative conditions. For example, under oxidative conditions and in the presence of fulvic acid, pure nanoparticulate Cu_*x*_S dissolution is complete within 15 to 20 days.^9^ In contrast, pure iron sulfide(s) and pyrite have zero-order reaction rates of 10^–8^ and 4 × 10^–11^ mol/s/m^2^ at circumneutral to acidic conditions, respectively,^56,57^ which indicates that a *d* = 200 nm FeS_2_ particle (similar size to what is detected in this study) would be stable for months under oxidative conditions. However, oxidative dissolution rates linearly increase with increasing chemical impurity in the lattice, further influencing the dissolution behavior.^58^ Thus, in the case of the proposed Cu_0.039_Zn_0.043_Fe_0.923_S phase, the dissolution kinetics will most likely resemble those of FeS or FeS_2_, which will also predict the concomitant release rate of Cu and Zn rather than that of pure CuS or ZnS.

We hypothesize that the presence of a well-defined Fe-sulfide phase in the hydraulic fracturing wastewater versus a poorly defined impure Fe-sulfide phase in the lake sediment can be traced back to differences in the genesis. The colloidal pyrite in the hydraulic fracturing wastewater was likely released during shale fracturing or shale dissolution,^15^ whereas metal sulfides in lake sediments are frequently formed by the precipitation of dissolved metals under reducing conditions with sulfide generated by sulfate-reducing bacteria.^59^ Iron sulfides generated through the latter process are good adsorbents for other metals^60^ and are thus more likely to produce impure metal-sulfide phases as they grow, which is in agreement with our results.

### 3.3 Mercury Sulfide in an Offshore Marine Sediment

**Figure 3:**
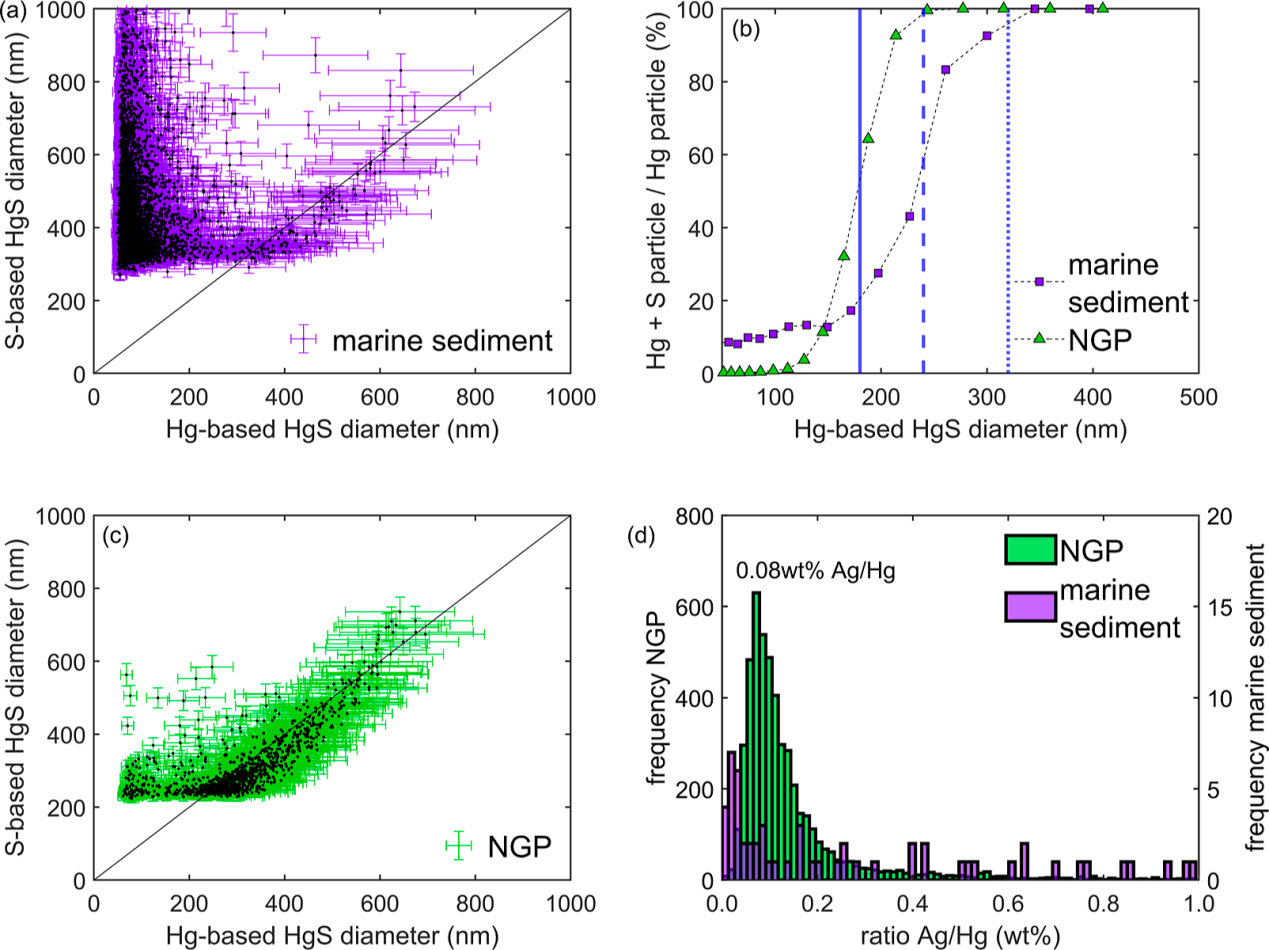
Metal sulfide colloids in a marine offshore sediment sample and sample obtained from industrial scale process (NGP). (a) S-based HgS diameter vs the Hg-based HgS for the marine sediment sample. (b) Relative frequencies of Hg (purple squares: marine sediment; green triangles: NGP) divided by the frequencies of particles in which at least Hg was detected. The vertical lines indicate colloid diameters at which S can be detected in 5, 50, and 95% of metal-sulfide colloids based on data derived from ZnS (Figure 1d). (c) S-based HgS diameter vs the Hg-based HgS for the NGP sample. (d) Ratios of Ag to Hg (in wt %) in the NGP sample and in the marine sediment. Sample NGP is displayed against the left axis, the marine sediment sample against the right axis. Comparable data for ratios of Pb to Hg are displayed in the Supporting Information (Figure S9).

A marine offshore sediment sample contained a large number of sulfide particles (*n* = 53,817) that could be traced back to multiple, distinct inorganic sulfide phases. Most of the S containing particles were associated with Cu, Zn, Fe, Pb, and Hg (Figure S4c). Fe or Hg were (by mass) the most important cations in individual S containing particles, as indicated by the Fe–S or Hg–S data scattered around the 1:1 lines (Figures 3a and S7). Cu and Zn occurred in shares of ∼ 3.0mol % Cu/S and ∼3.3 mol % Zn/S, respectively, which is slightly lower compared to the fractions found in the freshwater lake sediment sample. Accounting for the low ratios of Cu/S and Zn/S, we interpreted these data as an impure FeS phase that contained Cu and Zn as discussed for the lake sediment in the previous section.

The marine sediment contained a significant amount of Hg (∼200 mg/kg), which was reflected in *n* = 59,496 detected particles containing Hg, of which *n* = 5152 particles contained both Hg and S (Figure 3a). At Hg-based HgS diameter equivalents larger than ∼300 nm, all Hg in the marine sediment was associated with S, indicating that Hg was quantitatively associated with S (Figure 3b). Moreover, the increase at high Hg per particle mass in combination with the fact that above 400 nm the Hg versus S scatter along the 1 to 1 line, indicating the presence of HgS (Figure 3a). In the lower size range (Hg-based diameter *d* < 400 nm), the Hg and S signals were uncorrelated. None of the other detected elements were linearly correlated to Hg; therefore, the ratios of S/Hg > 1 in smaller particles likely resulted from an overestimation of the S signal toward the LOD as already discussed above and are likely HgS particles with their sulfide masses below the size limit at which S can be reliably quantified by sp-icpTOF-MS.

### 3.4 Impure Mercury Sulfide Particles in an Industrial Process Stream

The sample NGP was obtained from an industrial facility processing a resource of natural origin. We detected *n* = 2836 S containing particles, which represented ∼2% of the total detected particles in the sample. Most particles were associated with aluminum (Al), chromium (Cr), Fe, Ni, Cu, Zn, silver (Ag), Hg, and Pb (Figure S4d). For Hg-based HgS diameter equivalents larger than ∼300 nm, all Hg in the sample NGP was associated with S, indicating that Hg was quantitatively associated with S and occurred at a 1:1 ratio indicating the presence of HgS (Figure 3c,b).

XAS showed that Hg was predominantly present in the form of metacinnabar (β-HgS) in sample NGP (Section S10, Table S5 and Figure S8), thus confirming the 1:1 ratio of Hg and S determined by sp-icpTOF-MS. To explore the properties of the metacinnabar (HgS particles) on an individual particle level, the most frequently detected associated metals were plotted against Hg for all particles that contained S (Figure S10a–f). Of these secondary metals, Al, Cr, Fe, Ni, Cu, and Zn were poorly correlated to Hg, thus most likely attached as separate particles, whereas for Ag and Pb, a good correlation to Hg was obtained (Figure S10g,h). The slopes of the respective lines indicated the weight-by-weight ratio between the secondary metals and Hg. Ag occurred in trace amounts in the Hg–S particles, and the ratios of Ag/Hg in individual particles formed a narrow distribution around 0.08 wt % Ag per Hg (Figure 3d; comparable data with 0.11 wt % Pb per Hg ratio in Figure S9). Thus, Hg with minor additions of Ag and Pb (all chalcophilic elements) most likely formed a common sulfide phase rather than smaller Ag_2_(S) and Pb(S) being attached to larger HgS particles.

Here, we propose mass ratios in individual particles as a relevant metric, e.g., Ag/Hg or Pb/Hg to assess similarities between mercury sulfide in a marine sediment and the industrial process (Figures 3d and S9). A similar concept was proposed using the ratio of Ce to La to distinguish natural and engineered NPs^61^ or tracing the origin of PM2.5.^62^ Our data showed that the Ag/Hg or Pb/Hg ratios in HgS in the industrial process stream were well defined, whereas HgS in the marine sediment sample contained no clear trend. We explored whether chemical fingerprinting could be used to distinguish between HgS particles from either sample. Applying a KNN ML approach to classify HgS particles as previously introduced for copper sulfides, we find an accuracy of >99% using a total of 10 isotopes of S, Fe, Ag, Hg, and Pb (Figure S11 and details in Section S9). Only including isotopes of S and Hg reduced the accuracy to 65% (data not shown), indicating that impurities and not size distributions were key in distinguishing the origins of mercury sulfides with high levels of certainty.

## 4 Implications

This study demonstrated the use of sp-icpTOF-MS for characterizing the elemental stoichiometry of individual metal sulfide and metal phosphate colloids, where both the metal and S and P are quantified in each particle. Measurements of sulfur containing colloids in much larger populations of nonsulfidized particles, along with ML approaches, enable determination of the phase of individual particles. For example, distinguishing between copper sulfides of different stoichiometries. This method confirmed the presence of pyrite in hydraulic fracturing wastewater, likely released from the shale during the fracturing process. In contrast to the pure pyrite particles in hydraulic fracturing fluids, the metal sulfides found in most environments (e.g., sediments, oil, and gas processing streams) were impure. This has implications for studying the fate of metals in natural and anthropogenically impacted environmental systems. While laboratory studies frequently need to rely on single phases to investigate the reactivity, bioavailability, and toxicity potential of colloids, these single (pure) phases might be less present than anticipated, which has consequences for the reliability of predictions from those studies and could be factored into future study design on the fate of metal sulfides.

The approaches developed here also have implications for environmental forensics. The results indicated that silver occurred in mercury sulfide particles at a constant ratio in an industrial sample of geogenic origin. This constant ratio was absent in mercury sulfide originating from a marine sediment. The sp-icpTOF-MS methods developed here, in combination with ML, could potentially be used for mercury source tracking, even in the absence of isotopically labeled particles.^62^

In general terms, sp-icpTOF-MS data can be used to estimate the concentration and stoichiometry of individual metal phosphate and sulfide particles in an environmental sample along with any other trace metal impurities that may be present. The associations of different elements as a function of particle mass can be used to determine whether smaller particles are attached to larger carriers or occur as a single impure phase. This offers opportunities, for example, in combination with tests for acid volatile sulfides and simultaneous extract metals which offer quantitative information on sulfide associated metals in sediment, to assess the purity of individual metal (phosphates) in resource recovery processes,^63,64^ inorganic colloids formed in drinking water distribution systems,^65^ or for the improvement of determining the inhalation toxicity of PM2.5.^25,66^ PM2.5 which potentially penetrates into lungs, enters the bloodstream and causes adverse effects, also contains metal phosphates and sulfides.^23,25,66,67^ Thus, determining the elemental composition of individual metal phosphates or sulfides and whether a potentially toxic metal occurs in an amalgam or as a pure phase can inform on its bioavailability and thus toxicity potential.

## Supporting Information Available

Contains information on sp-icpTOF-MS operation, post processing rules for particle detection and quantification, preparation of synthetic colloids, environmental sample preparation, occurrence of lead phosphate in contaminated soils, and XAS data acquisition and evaluation (PDF)
