# Supplementary material for: Characterizing the Stoichiometry of Individual Metal Sulfide and Phosphate Colloids in Soils, Sediments, and Industrial Processes by Inductively Coupled Plasma Time-of-Flight Mass Spectrometry
Source: Environ Sci Technol. 2024 Jun 25;58(27):12113–22. doi: 10.1021/acs.est.3c10186 (PMC11238586; doi:10.1021/acs.est.3c10186)
Supplement: Supplementary file 1 — es3c10186_si_001.pdf [file es3c10186_si_001.pdf]

Supporting Information for

Characterizing the stoichiometry of individual metal sulfide and phosphate colloids in soils, sediments and industrial processes by inductively coupled plasma time-of-flight mass spectrometry

Jonas Wielinski*, Xiaopeng Huang and Gregory V. Lowry

Department of Civil and Environmental Engineering, Carnegie Mellon University, Pittsburgh PA, 15213, USA

*Corresponding author: Jonas Wielinski, jwielins@andrew.cmu.edu

Table of contents

S1. Isotope detection requirements for elements with multiple accessible isotopes S19

S2. Self-aspirating nebulization versus peristaltic pump sample introduction S20

S3. Requirement of lead signal notching for correct stoichiometry in Pym S21

S4. Sp-icpTOF-MS external calibration S21

S5. Preparation of nano and macro-phases for method validation S22

S6. Soil, sediment, industrial and hydraulic fracturing wastewater sample preparation S23

S7. Additional case study: Formation of nano scale lead phosphate in P treated shooting range soil S24

S8. Characterization of the P signal towards the LOD S25

S9. Machine learning for distinguishing copper sulfides and mercury sulfides S26

S10. X-ray absorption spectroscopy (XAS) data collection, evaluation, and results S27

S11. Supporting Tables (contains 5 tables) S29

S12. Supporting Figures (contains 25 figures) S35

S13. References S37

S1. Isotope detection requirements for elements with multiple accessible isotopes

The appropriateness of different approaches to distinguish between the (dissolved) background signal and particle events in single particle detection by icpTOF-MS is under debate.[1–3] The challenge is to apply sufficiently strict thresholds without losing particles that are wrongly classified as dissolved background. Here, we followed the approach of Tanner and Günther (2009) [1] (details in Materials and Methods and eq. 1), but introduce additional restrictions to reduce the occurrence of false positive particle events, *i.e.*, an event is erroneously registered as a particle. For illustration, the particle size distribution (PSD) of sample Pym was determined by DLS and sp-icpTOF-MS in no-gas mode. DLS indicated mode particle sizes of ~50 nm or ~60 nm as number or volume-based PSDs (Figure S21). Three different sp-icpTOF-MS based PSDs were calculated. One with all particles that contained at least ^{208}Pb (red bars), a second one with ^{206}Pb, ^{207}Pb and ^{208}Pb (violent bars) and a third one with ^{204}Pb, ^{206}Pb, ^{207}Pb and ^{208}Pb (yellow bars). When only ^{208}Pb was required, smaller diameters were found compared to when multiple isotopes were required. The DLS and TOF PSD matched best, when the detection of all four available isotopes was required. Tanner and Günther (2009)[1] assume the background signal to be Poisson-distributed, whereas other research has argued that compound Poisson distribution is more appropriate to accurately separate the (dissolved) background from gold nanoparticles (NP) particle events.[2] Gold-NP are however, a special case, since only one isotope (^{197}Au) is available for analysis. The most important analyte metals in this study (Fe, Cu, Zn, Ag, Hg, Pb) have more than one accessible isotope, which is exploited for post-processing of particle events as follows.

It was considered that the sum of all registered particle events p of one isotope of an element with multiple (or only one) detectable isotopes consisted of true positive (p^+) and false positive (p^-) counts (eq. S1). The true positive (p^+) counts are those in which ions of a particle hit the detector, whereas the false positive (p^-) counts are those of dissolved background that were erroneously labeled as a particle. The goal of all particle detection methods is to maximize p^+ and minimize p^-.

$$p = p^+ + p^- \qquad \text{(eq. S1)}$$

Eq. S1 can be rearranged to calculate an isotope specific false positive rate (q^-) (eq. S2). The false positive rate (q^-) is the ratio of the false positive particle events and all particle events of the selected isotope.

$$q^- = \frac{p^-}{p} = \frac{p-p^+}{p} \qquad \text{(eq. S2)}$$

It is reasonable to assume that the occurrence of false positives is random and thus independent in different isotopes of the same element (but also of isotopes of differing elements). Therefore, including multiple isotopes of the same element (or of differing elements that occur in the same particle) will result in an overall

false positive rate (q_n^-), where n is the number of isotopes required for the successful registration of a particle (eq. S3). Since q^- is $0 < q^- < 1$ (in most cases), and n = 2, 3, .., it follows that $q_n^- < q^-$ and thus including more isotopes reduces the false positive rate.

$$q_n^- = (q^-)^n \qquad \text{(eq. S3)}$$

The true false positive rate cannot be determined; however, it can be estimated by minimizing eq. S4 and selecting n to become i and j that must be $i \neq j$. The parameters i and j represent the number of isotopes included in the analysis.

$$\arg\left(\min\left(1 - \frac{p_i(q^-)^j}{p_j(q^-)^i}\right)\right) \qquad \text{(eq. S4)}$$

Data of ~16,000 Pym particles from different measurements was gathered (Figure S22a), and i and j were varied from 1 to 4. This resulted in $q^- = 0.49 \pm 0.22\ (1\sigma)$ (or q_n^- = 0.058 ± 0.002) (Figure S22b). To estimate whether these numbers are reasonable, we generated a dataset for comparison using TOF and DLS data. It was assumed that the DLS PSD was more accurate compared to the TOF PSD and all particle events with d < 30 nm, *i.e.*, below the lower size end of the DLS PSD were false positives (Figure S21). A cutoff of 30 nm is also in agreement with Pym particles visible in electron micrographs (Figure S1a-c). The resulting false positive rates fit well to those determined by eq. 4 (Figure S22b) and thus suggesting that including more isotopes will reduce the amount of false positive counts. Therefore, whenever possible multiple isotopes were required for a particle event to be considered for analysis, *e.g.*, $^{204,206\text{-}208}$Pb for Pym and $^{206\text{-}208}$Pb, ^{63,65}Cu and ^{64,66,68}Zn for soil and sediment samples (Table S3). This approach does not recognize the differences in isotopic abundances and is relatively restrictive. Therefore, it may not be suitable to determine accurate particle number concentrations, but potentially more accurate at lower particle sizes as shown by comparison to DLS data.

S2. Self-aspirating nebulization versus peristaltic pump sample introduction

Conventional sample introduction for single particle ICP-MS (TOF or Q(QQ)) is often facilitated using a peristaltic pump. Prior research showed that self-aspirating nebulizers can, while operating at significantly reduced flow rates, increase the transport efficiency (solution to detector) and thus reduce sample consumption. In this research, we were not limited by the available sample volume. However, utilization of a self-aspirating nebulizer eliminated oscillations induced by the peristaltic pump in the presence of dissolved background, which potentially affected particle detection and size determination. For example, calculating the single sided amplitude spectrum (|P1(f)|) of a dissolved Au signal at different rotational pump speeds (Figure S18), showed that the pump induced an artifact into the signal (see black arrow, Figure S18).

Sp-icpTOF-MS data of sample Pym was collected in O_2 mode for the parallel detection of P and Pb (identical sample, acquisition time 360.4 s, dwell time 2 ms, tuned to $^{31}P^{16}O^+$) with either the conventional sample introduction setup (peristaltic pump) or the self-aspirating nebulizer but otherwise identical acquisition parameters (both systems are described in the materials and methods section of the main text). Based on the Pb signal, around 4000 or 6000 particles were detection with or without usage of the peristaltic pump, respectively, meaning an increase of 50% (Figure S19a). At the same time, the particle size distribution recorded using the self-aspirating nebulizer was narrower and shifted to lower intensities, which resulted from a reduced background signal (Figure S19b). Pb, with high sensitivity and low limit of detection (LOD) is relatively uncomplicated to detect. P containing particles are, on the other hand, more difficult to detect. Here, using the self-aspirating nebulizer allowed detecting almost 4 times more particles compared to the standard configuration (Figure S20a), even though the background signals were almost indistinguishable and the LOD [1] calculated from eq. 1 was only ~24% higher when the standard sample introduction configuration was used (0.1062 versus 0.1312 fmol/particle). These results showed that stabilizing the sample introduction system was a key parameter to successful quantification of P (and S) containing colloids by sp-icpTOF-MS.

S3. Requirement of lead signal notching for correct stoichiometry in Pym

Sp-icpTOF-MS data in O_2 reaction mode was collected on Pym without using a notch filter. We computed the Pym diameter from the Pb and P signal and plot them against each other (blue squares, Figure S23). An ideal measurement would fall on the dashed, black line. The displayed data separated into two parts with different slopes. The lower size end (Pb based diameters ~90-220 nm) was characterized by a near-zero slope, whereas the upper size end (d = 220-350 nm) exhibited a slope exceeding the dashed line representing the Pb to P ratio in ideal Pym. These data indicated that the P mass per particle was being overestimated. We hypothesize that for smaller particles (d < 220 nm) the P detection was inaccurate because the particles were close to the P limit of detection (LOD), whereas for larger particles, the Pb signal intensity was higher than the linear range of the detector. To reduce the amount of Pb ions reaching the detector, one notch filter channel was set to m/z 209 to reduce the Pb sensitivity. This reproduced a more accurate stoichiometry of Pb and P in Pym as discussed in the results section of the main text.

In oxygen reaction mode, isotopes can be recorded on-mass (*e.g.*, assuming a charge of 1 and no reaction of the analyte isotope with oxygen) or after a mass-shift as done for P and S in this research. Other elements, such as Fe or As can also be measured after a mass-shift. In both cases, the sensitivity is even higher after a mass-shift compared to on mass measurements. However, for the same reason as discussed above for Pb, we selected the isotope of lower sensitivity (*e.g.*, 298 cps/ppb for $^{54}Fe^+$ versus 709 cps/ppb for $^{54}Fe^{16}O^+$) so

that similar masses of metals, P and S in particles fall within the linear range of the detector. In addition, this allows us use existing isotope databases rather than establishing methods for metal detection after mass-shift.

S4. Sp-icpTOF-MS external calibration

External calibrations were recorded using three multi element ICP standard solutions covering 71 elements and an additional Hg standard solution using eight calibration points between 0.01 and 20.0 ppb (Environmental Express), P using five calibration points between 0.186 and 1860.0 ppb (TraceCert, Supelco, MilliporeSigma) and S using seven calibration points between 0.100 and 5000.0 ppb (TraceCert, Supelco, MilliporeSigma). The best R^2 obtained for P and S were 0.9995 and 0.9927, respectively. Other values were not significantly lower. The standard deviations of the slope and y-axis intercept of the calibrations were used to calculate error bars where isotopes were plotted against each other. The Fe mass/particle was calculated using ^{54}Fe (5.85% abundance). At mass/charge 54, the calibration solutions also contained ^{54}Cr (4.34% abundance). Since both abundances were of similar quantity, the ^{54}Fe-derived mass was corrected by 0.57=0.0585/(0.0585+0.0434). The same type of correction was applied to ^{204}Pb to compensate for ^{204}Hg. In all other cases, masses were calculated using an isotope dominant at the relevant mass/charge ratio. The transport efficiency (typically around 4%) and particle masses (in g) were determined according to Pace et al.[4] Gold nano particles (d = 40 nm, NanoXact, Nanocomposix) were used as particle size standard.

S5. Preparation of nano and macro-phases for method validation

Pyromorphite (Pym), a member of the Pym-mimetite (Mim) solid solution, and ZnS were selected as representative P or S containing colloids. A multi-inlet vortex mixer (MIVM) with four ports designed for flash nano-precipitation [5,6] was used mix educts in aqueous solution. Injection of educts was done manually at ~30 mL/min with four 1 mL luer lock (LL). Precipitates were dripped directly into vigorously stirred 6 mg/mL sodium citrate tribasic (ACS grade, Sigma-Aldrich) to prevent aggregation. All described solutions were prepared in NanoPure deionized water (18.2 MΩ×cm, Merck Millipore, USA) purged with N_2 for at least 1 h prior to usage to remove dissolved oxygen (DO). The injection solutions and chemicals are tabulated (Table S4). The size of precipitates was determined by dynamic light scattering (DLS, Zetasizer Nano, Malvern: d = 30 – 100 nm, Figure S12). For DLS measurements fresh synthesis products were diluted in DI water and measured without further processing.

The synthesis of nano ZnS has been reported by a number of studies,[7] and is thus not discussed in detail here. Briefly, Zn(II) (from zinc nitrate hexahydrate, ACS grade, Sigma-Aldrich) solutions and S(-II) (from

sodium sulfide nonahydrate, ACS grade, Sigma-Aldrich) were mixed in the MIVM with a rate of ~30 mL/min to precipitate ZnS (sample ZnS, bimodal size distribution, DLS d = 40-300 nm) (Figure S12).

For chalcopyrite synthesis, 16 mmol iron(III) acetylacetonate (5.6507 g), 16 mmol copper(I) iodide (3.0472 g), 60 mL 1-dedecanethiol and 20 mL 1-octadecene were mixed in a 500 mL round-bottomed flask. The flask was sealed with a rubber septum, which was punctured by two needles. The mixture was constantly stirred under nitrogen flow at room temperature for 60 min. Successively, the temperature was increased to 100 C and 230 C in 60 min intervals. The product was allowed to cool to room temperature and washed three times using acetone.

Synthesis products were washed prior to sp-icpTOF-MS, X-ray diffraction (XRD) and transmission electron microscopy (TEM) analyses. For washing, 1.5 mL of synthesis product were centrifuged at 16.1k g for 10 min and 1.46 mL of the supernatant was replaced by DO free DI water, which was repeated three times. This process led to partial aggregation of primary particles. TEM micrographs were recorded on samples Pym and ZnS using a FEI Tecnai F20 Super-Twin set to 200 kV high tension for imaging in bright field on a high-resolution Gatan Rio CCD Camera. Electron micrographs of sample Pym showed partially aggregated particles between 30 and 60 nm of primary particle size (Figure S1a-c), whereas micrographs of ZnS mostly showed mostly strongly aggregated sub 10 nm particles forming clusters of up to 200 nm (Figure S1d-e).

XRD patterns of Pym, ZnS, $CuFeS_2$ and Pym-mc (macro crystalline Pym, preparation below) were recorded on an Empyrean (Malvern Panalytical) equipped with a Cu target. Results showed that the nano sized precipitates were Pym (Figure S14). XRD further showed that ZnS was poorly crystalline sphalerite (Figure S13c). The nano chalcopyrite was confirmed to be of the chalcopyrite crystal structure by XRD (Figure S15).

Natural covellite and chalcocite minerals were kindly provided by the Department of Earth Sciences at Stanford University, CA (USA). Individual crystals were separated from the host rock and ground using mortar and pestle. The fine powder was placed in DI water, tip sonicated, passed through a 1 µm syringe filter, diluted in DI water and measured by sp-icpTOF-MS. Naturally occurring pyrite was obtained from Alfa Aesar and prepared identically.

Macro crystalline Pym (sample Pym-mc) was prepared as previously reported. [8] Briefly, solutions of $Pb(NO_3)_2$, KH_2PO_4 and KCl were mixed in a beaker initially containing 100 mL of constantly stirred DO free DI water at Q = 1.0 mL/min using a peristaltic pump over 100 min. Large precipitates settled immediately. The precipitates were collected on a 0.45 µm filter, rinsed with acetone and dried under vacuum at 50 °C overnight.

S6. Soil, sediment, industrial and hydraulic fracturing wastewater sample preparation

A Pb contaminated soil sample was obtained from a shooting range in Switzerland. The soil was air dried and sieved to $d < 2$ mm. The Pb concentration was 1858 ± 3 ppm (determined by X-ray fluorescence spectroscopy on a XEPOS+, SPECTRO Analytical Instruments). 10 g of soil were added into a 50 mL centrifugation tube and treated with 2 mg P (from H_3PO_4) [9] diluted in 2.5 mL of DI water to reach a gravimetric water content of 25%. The top of the tube was wrapped with para film into which holes were poked to enable gas exchange, yet to minimize evaporation. Around 200 µL DI water were added weekly to counteract evaporation. The sample was stored in the dark at room temperature for 134 days before measurement.

An urban subaquatic lake sediment was collected from North Park Lake (WGS 84 Web Mercator coordinates 40.602, -80.004), 18 km north of Pittsburgh, PA (USA). Around 15 L of sediment were collected and stored in an airtight container at 8 °C. An offshore marine sediment containing ~200 ppm of Hg was obtained (Hg concentration was determined using a Brooks-Rand Model MERX Hg analyzer after sediment digestion according to EPA Method 1631). Previous research indicated that Hg occurs as metacinnabar (*β*-HgS) in marine sediments, which was assumed in this study.[10]

For sp-icpTOF-MS measurements of soil and sediment, 200 mg of untreated or treated soil or 1 g of sediment were added into a 14 mL centrifugation tube. 10 mL of DO free 10 mM NaCl solution were added and vigorously shaken for 30 min, centrifuged (9500 rpm, 8 min) and decanted. 10 mL DO free DI water was added, the sample was shaken, centrifuged and decadent. This was repeated 2-times. Finally, 10 mL of DO free DI water were added and additional 200 µL of a 0.1% CMC were added. The samples were vigorously shaken for 30 min and treated by bath sonication for another 30 min until less than 5 min before sp-icpTOF-MS sample uptake to minimize colloid dissolution after dilution in DO containing DI water. Larger colloids were allowed to settle. For each sample, 2 µL of sample were added to 10 mL of DI water and shaken until measurement.

Hydraulic fracturing wastewater including (suspended) solids was obtained from an unconventional natural gas extraction field located in the Midland Basin, Wolfcamp formation, Midland, TX (USA). 50 mL of sample were added to a 50 mL centrifugation tube and centrifuged at 9000 rmp for 8 min. The supernatant which contained a large share of hydrocarbons was decanted. This step was repeated 3 times. 1 µL of sample was added to 10 mL of DI water, tip sonicated for 1 min and shaken until measurement.

A sample (called NGP) was obtained from a full-scale industrial facility processing a natural resource which contained around 2000 ppb of total Hg in a water/organic mixture (determined by a cold vapor atomic

fluorescence spectroscopy (CVAFS)-based mercury analyzer, Brooks Rand Instruments). 500 mL of sample NGP were passed through a 0.1 µm polyvinylidene fluoride (PVDF) membrane filter (Merck Millipore), which resulted in a dense, brownish filter cake. For sp-icpTOF-MS measurements, 1/4 of the filter was added to 25 mL of DI water, bath sonicated for 30 min and diluted 1 to 20 less than 5 min before sp-icpTOF-MS sample uptake.

S7. Additional case study: Formation of nano scale lead phosphate in P treated shooting range soil

Application of sp-icpTOF-MS readily identified changes in the chemical compositions of Pb colloids after soil phosphate treatment for Pb immobilization. In the untreated soil, ~22% of lead containing particles were not associated with other elements (Pb-only particles), whereas through P treatment this number strongly declined (Figure S4e). These associations are consistent with prior reports that bullet derived Pb in untreated shooting range soil occurs as cerussite ($PbCO_3$), litharge (α-PbO), hydrocerussite ($Pb_3(CO_3)_2(OH)_2$), kintoreite ($PbFe_3(PO_4)(PO_3OH)(OH)_6$), or is associated with soil organic carbon.[11–13] The occurrence of lead-only particles in untreated soils is consistent with these reports because C and O forming most of the above mentioned minerals are not discernable in the sp-icpTOF-MS. It has also been reported that weathered Pb frequently occurs sorbed to Fe-oxides and to manganese (Mn) oxide.[14,15] The majority of the remaining Pb-containing particles were associated with Fe and Mn, elements that suggest weathered Pb-bullets or geogenic Pb (Figure S4e).

The sp-icpTOF-MS data also indicated an incomplete crystallization reaction between the Pb and the added phosphate (Figure S25). The P/Pb ratios were higher than the theoretical value for Pym and most of the particles were small, likely due to the relatively short (143 d) reaction time used in this study.[16] Longer reaction times would potentially lead to larger Pym particles with the expected stoichiometry as shown by numerous other studies.[9,16–18].

Examination of elemental ratios in particles before and after adding phosphorus provided insight into the reactive pool of Pb in the soil. For example, the addition of P frequently leads to a significant decrease in the Mn/Pb ratio in the Pb-Mn particles (Figure S16a), which suggests that Pb sorbed onto Mn-oxides is a labile, readily available Pb-pool in the soil. The persistence of Mn in those particles, albeit at a lower concentration, also suggests that nano scale Pym crystals grew on Mn-oxide interfaces. In contrast, the Sb/Pb ratio of 3-4% remained unchanged by the presence of P (Figure S16 and Figure S17). In some Swiss made bullets, the Pb-core contains 2-5 wt% Sb,[19] thus in good agreement with the detected Sb/Pb ratio which indicated that the Sb-Pb particles were likely unreacted bullet fragments that were resistant against reaction with the added phosphorus.

These particle specific data are consistent with the conceptual model that addition of P to Pb contaminated soil promoted the formation of nano-scale Pb-P particles that were likely Pym associated with other particles,[9,17] and the precipitated Pb was preferentially derived from the labile Pb pool associated with Mn-oxides as opposed to more stable pools such as bullet fragments. The formation of small heterogeneous nano-polycrystalline Pb-phosphates will potentially lead to enhanced reactivity and bioavailability compared to macro-crystalline stoichiometric Pym.

S8. Characterization of the P signal towards the LOD

Toward the calculated P LOD (Table S3), the measured P concentration increased (Figure 1a). This could be because the particles are enriched in P relative to Pb, or because the instrument sensitivity to P increases. However, Pb concentrations can be determined much more accurately in this size range (Table S3) and are thus be considered more accurate than P concentrations. To better characterize the P-signal towards the LOD, the $Pb_5(PO_4)_{1.5}(AsO_4)_{1.5}Cl$ member of the Pym-mimetite solid solution (here referred to as Pym-Mim) was measured by sp-icpTOF-MS. The sensitivity of As was higher compared to P and the LOD was lower, which allowed measurement of smaller aggregates (Table S3). The molar ratio of P/As *versus* the Pb based diameter should be constant yet followed an exponential decay (Figure S2a). Therefore, the increasing P/As ratio closer to the P LOD in smaller particles was an artifact of the measurement instead of the synthesis and the exponential decay function can be used to correct the P signal of Pym. The corrected Pym fell around the 1:1 line and the slope was 0.93 ± 0.2 (Figure S2b). However, the origin of the increase of the $^{31}P^{16}O^{+}$ signal towards the LOD remains unknown, but we assume that the same mechanism is also responsible for the increase of the S signal towards the LOD (Figure 1c).

S9. Machine learning for distinguishing copper sulfides and mercury sulfides

We used the k nearest neighbors (KNN) classifier implemented in the scikit-learn package in Python3 (‘neighbors.KneighborsClassifier’).[20] Data was prepared by extracting the $^{32}S^{16}O$, ^{63}Cu, ^{65}Cu, ^{54}Fe, ^{56}Fe, and ^{57}Fe (a total of 6 isotopes or columns) from datasets of CuS, Cu_2S, and $CuFeS_2$, which was labeled. Multiple isotopes of Cu and Fe were used to increase the amount of information in the model. Masses were converted to log-space and scaled for preprocessing (‘preprocessing.scale’). Missing particle masses below the LOD were set to zero. Any row that contained four or more zeros was removed. This ensures that all particle events considered in the analysis are the target particles and avoids misclassifying copper sulfides without iron, *e.g.*, CuS and Cu_2S because only three isotopes can be detected for these particles. Using rows with only one or two entries would not be able to distinguish between Cu_xS particles and $CuFeS_2$ particles. Applying this rule to the analyzed particles enables distinguishing between iron sulfides, copper sulfides or

iron-copper sulfides. Requiring fewer non-zero entries also violates our rule that multiple isotopes should be detected where possible (SI Section S1). No other particle selection according to the size was made to remove values close to the LOD or those suffering from non-linearity. This preprocessed dataset was randomly split with training and test set sizes of 75% and 25%, respectively (‘train_test_split’). The training set was used to train the classifier varying the number of neighbors finding n=6 as the optimal value. The test set was used to predict the accuracy of the classifier typically achieving overall values around 98.5%.

Classification was the most promising approach to distinguish between these three phases [21] compared to (linear) regression for two reasons. First, in the larger size ranges we observed stoichiometry-dependent non-linearity effects in elemental ratios *i.e.*, the higher the ratio of Cu to S, the more the Cu mass was underestimated relative to the S mass (Figure S3). Second, the number of relevant isotopes depended on the particle stoichiometry, *e.g.*, Cu and S isotopes for CuS *versus* Cu, Fe, and S isotopes for $CuFeS_2$.[21]

The above-introduced approach was used to distinguish HgS particles in the NGP sample from those in the marine sediment. Particle events in the training and testing datasets were required to contain $^{32}S^{16}O$, ^{200}Hg and ^{202}Hg. In addition, ^{54}Fe, ^{56}Fe, ^{57}Fe, ^{107}Ag, ^{109}Ag, ^{206}Pb and ^{208}Pb were included in the analysis. The prediction accuracy was ~99.3%.

Both problems were also addressed using logistic regression (LR) as an alternative to KNN for classification problems. In both cases, KNN outperformed LR (Figure S24) and thus, KNN results were chosen for presentation in this publication. This has been previously observed for single particle time-of-flight derived data.[21]

S10. X-ray absorption spectroscopy (XAS) data collection, evaluation and results

X-ray absorption spectroscopy (XAS) data was collected at the mercury (Hg) L_{III} edge (12.284 keV) at beamline (BL) 11-2, at Stanford Synchrotron Radiation Lightsource (SSRL) operated by the Stanford National Accelerator Laboratory (SLAC) in Menlo Park, CA. BL 11-2 is a high-flux station generating synchrotron radiation using a 26-pole 2.0-Tesla wiggler and a double-crystal, variable-exit geometry liquid nitrogen (LN) cooled Si(220) monochromator set to ϕ = 90° for energy selection. The monochromator was detuned to 70% relative to the maximum intensity at 12456 keV (the arithmetic mean of all energies at which data was recorded) to minimize the contribution of higher harmonics to the x-ray beam. Slits were used to cut the beam to 3.5 × 1 mm^2 in horizontal and vertical direction. Data was recorded with a 10-eV step from 12.054 keV to12264 eV, with a 0.35 eV step to 12314 eV and up to k = 12.2 $Å^{-1}$ with Δk = 0.05 $Å^{-1}$. Scan time was ~31 min. Duplicate scans were performed on samples and references. Quick scans (~1

min/scan) were performed around the edge to detect possible changes in the Hg speciation resulting from beam irradiation, but no radiation induced changes were detected.

Three ionization chambers were used to record data of model compounds and a SnHg alloy foil for energy alignment in transmission mode. Data on sample NGP (filtered suspended matter from an organic solvent/water mixture obtained from a full-scale industrial facility processing a natural resource) was recorded in fluorescence mode. Samples were placed under vacuum in a LN-cooled cryostat at T = 77 K. For fluorescence detection a 100-channel germanium detector was set up at 90° relative to the incoming beam and the sample was rotated to 45° towards the detector. A gallium filter, Soller slits and one layer of aluminum foil were placed between the sample and the detector to reduce scattering and fluorescence contribution from lighter elements, especially iron. Of the 100 detector channels, 80 channels were recording fluorescence signals. In 7 of these channels, the incoming and outgoing count rates were not well correlated ($r^2 < 0.95$) or the recorded signal in the region of interest exceeded the linear range of the detector. These channels were excluded from data evaluation leading to a total of 73 included channels. This part of data pretreatment was done in Matlab.

For fingerprinting and linear combination fitting (LCF), spectra were loaded into the ATHENA software code. Spectra were normalized and converted to k-space using the implemented AutoBK algorithm. The following model compounds were recorded and compared: metacinnabar (β-HgS), Hg-cystine (Hg-Cys), metallic Hg, Hg-bound to polysulfide, and Hg-$Na_2S_2O_3$. LCF to the NGP XANES spectrum resulted in 77% β-HgS and 23% Hg-Cys, whereas LCF to the NGP EXAFS spectrum resulted in 79% β-HgS and 21% Hg-Cys (Figure S8ab). Other model compounds were not relevant in the LCFs, and thus we concluded that β-HgS was the predominant component in the NGP sample and minor shares of Hg were present as bound to cysteine.

For an additional characterization, shell fittings were performed to obtain a quantitative comparison between β-HgS and NGP using the ARTEMIS software code. Fits recovered the expected parameters well (Table S5). Both, the coordination number (CN), and the Hg-S bond lengths (R) match those obtained from the literature.[22] The values of all other fitting parameters (amplitude reduction factor, energy shift between the calculated path and the measured spectra and the mean square displacement (σ^2)) were reasonable and of low uncertainty. The quality of the fit to the β-HgS spectrum was slightly better than the quality of the fit to the NGP spectrum as indicated for example by the lower uncertainty in σ^2 and R and the better agreement of R between β-HgS and the theoretical value compared to NGP. This eventually resulted from the presence of a minor Hg phase other than β-HgS in NGP as shown by the LCF results.

In conclusion, LCF results and shell fitting results indicated that Hg in the NGP sample was predominantly present as β-HgS.

S11. Supporting Tables

Table S1. All elements that were considered in this study and post processing rules for particle detection and quantification. * ^{63}Cu was used due to the interference of ^{65}Cu with $^{32}S^{16}O^{1}H^{+}$ and other S and O containing species. Only Cu particle events were included where the ratios of the masses calculated from ^{63}Cu and ^{65}Cu ranged between 0.9 and 1.1. ** The ratio of ^{64}Zn and ^{66}Zn was used to determine whether a particle event was included in the evaluation. The lower part of this Table contains relevant computations to explain the inclusion/exclusion rules for P and S. To detect P ($^{31}P^{16}O$), m/z 47 must be detected, but not m/z 46 and 48. These rules eliminate Ti interference given that ^{48}Ti is the most abundant and sensitive isotope and the absence of ^{46}Ti confirms the absence of Ti. To detect S ($^{32}S^{16}O$), m/z 48 must be detected, but not m/z 46 and 47 to avoid misclassifying Ti in particles as S. Low relative abundance of ^{46}Ti and ^{47}Ti compared to ^{48}Ti makes distinguishing S from Ti potentially more challenging than distinguishing P from Ti. However, due to the significantly higher sensitivity for Ti compared to S, the lower abundance of ^{46}Ti and ^{47}Ti can still be easily detected at similar particle sizes to distinguish between S and Ti bearing particles.

Element	m/z that must be present	m/z that must not be present	m/z used for quantification
Al	27		27
Ti	46, 47, 48		46
P	47	46, 48	47
S	48	46, 47	48
V	51		51
Cr	52, 53		52
Fe	54, 70		54
Mn	55		55
Ni	58		58
Co	59		59
Cu	63, 65		63 *
Zn	64, 66, 68		66 **
Ga	69		69
As	75, 91	90	75
Se	78		78
Rb	85		85
Sr	88		88
Cd	112		112
Sb	118		118
Ba	137		137
La	139		139
Ce	140		140
Pr	141		141
Pt	194, 195, 198		195
Hg	200, 202		202
Pb	204, 206, 207, 208 for Pym or 206, 207, 208 for soil samples		208

Relevant computations for inclusion/exclusion rules of elements P and S						
m/z	Ti (% abundance)	Sensitivity (cps/ppb)	Abundance × sensitivity for Ti	P or S abundance	Sensitivity (cps/ppb)	Abundance × sensitivity for P or S
46	8.3	983	8158.9	0	-	-
47	7.4	946	7000.4	100% (P)	116	11600
48	73.7	11790	868923	95.02% (S)	110	10452.2

Table S2. icpTOF-MS instrument parameters that were tuned to either $^{31}P^{16}O^+$ (for measurements of P and/or S) or $^{32}S^{16}O^+$ for measurements of S and in no-gas mode. In no-gas mode, the nebulizer flow was optimized for maximum $^{115}In^+$ sensitivity with the conditions of $^{140}Ce^{16}O^+/^{140}Ce^+$ (oxide ratio) < 5%. The oxide ratio was not considered in O_2 reaction mode.

Parameter	No gas mode standard sample introduction	No gas mode with microflow and linear spray chamber	O_2 reaction mode: $^{31}P^{16}O^+$	O_2 reaction mode: $^{32}S^{16}O^+$
Torch horizontal position	-1.06	-0.73	-0.74	-0.84
Torch vertical position	-1.06	-0.27	-0.31	-0.28
Nebulizer flow	1.0552	0.9957	1.0485	1.0279
Extraction lens 2	-238.77	-210.15	-181.58	-165.7
CCT focus lens	4.81	2.67	-9.46	-10.68
Angular deflection	-467.47	-419.17	-409.41	-379.63
CCT entry lens	-156.34	-106.36	-192.03	-191.5
D1 lens	-199.1	-199.21	-190.35	-191.24
Focus lens	19.25	19.43	16.23	15.76
Quad entry lens	-30.33	-26.93	-22.52	-21.22

Table S3. Typically measured sensitivities (cps/ppb) of selected isotopes including their ratios to P (Pb) or S (all others). Unless indicated otherwise, all isotopes were measured on-mass based on reported in existing databases. The limits of detection (LODs) were calculated according to eq. 1 from Tanner et al.[1] Cu, Zn, Hg and S LOD were calculated based on the background data of the urban lake sediment, P and Pb (with and without notch filter) LOD were calculated based on the treated contaminated soil data. Values varied marginally between instrument runs. * These sizes were calculated based on the LOD (fmol/particle) derived from Tanner and Günther (2009)[1] according to eq. 1. These do not represent the sizes at which the accurate stoichiometry is recovered (see section 'Detection of metal phosphate or sulfide stoichiometry in colloids of known composition and classification by machine learning' in the main text).

Isotope	Sensitivity (cps/ppb)	Relevant ratio	Limit of detection (LOD) in fmol/particle
In oxygen reaction mode			
^{54}Fe	298	~2.7	0.008146
^{54}Fe(^{16}O)	709	~6.5	0.003211
^{63}Cu	3335	~30	0.000286
^{65}Cu	1763	~16	0.001114
^{64}Zn	4443	~40	0.000616
^{66}Zn	1588	~14	0.000523
^{75}As	232	-	0.008924
^{75}As(^{16}O)	1042	-	0.002062
^{202}Hg	852	~7.8	0.001642
^{204}Pb	1581	~14	0.0008
^{208}Pb	4800	~41	3.42×10^{-5}
^{204}Pb (notched)	95	~0.8	0.005723
^{208}Pb (notched)	329	~2.8	0.001743
^{31}P(^{16}O)	116	-	0.1062 (= 110 nm for Pym*)
^{32}S(^{16}O)	110	-	0.1177 (= 110 nm for ZnS*)
Selected isotopes in no-gas mode for comparison to O_2-reaction mode			
^{54}Fe	1097	-	0.03514
^{63}Cu	6017	-	0.00060
^{65}Cu	2920	-	0.00086
^{64}Zn	3809	-	0.00138
^{66}Zn	2249	-	0.00170
^{202}Hg	3400	-	0.01082
^{208}Pb	21780	-	0.00009

Table S4. Samples that were analyzed in this study. Samples Pym, Pym-Mim, Pym-mc, ZnS and chalcopyrite ($CuFeS_2$) were synthesized. Other samples (untreated soil, treated soil, urban lake sediment, marine off-shore sediment, and NGP) were analyzed to show and discuss the capability of the presented sp-icpTOF-MS method detecting P and S. Fields labeled 'not available' (N/A) refer to properties or parameters that were not applicable for this specific sample. * Particle number concentrations (PNCs) represent total PNCs, *i.e.* were calculated using all isotopes that were detected by sp-icpTOF-MS in the dilutions.

Sample ID	Port A	Port B	Port C	Port D	Stabilizing agent	DLS size (nm)	TOF data acquisition time	PNC (particles/mL)*
Pym	10 mM $Pb(NO_3)$	6 mM K_2HPO_4	2 mM KCl	DI water	6 mg/mL Na_3-citrate	30-100 nm	12 min	3.87E+04
Pym-Mim	10 mM $Pb(NO_3)$	3 mM K_2HPO_4, 3 mM Na_2AsO_4	2 mM KCl	DI water	6 mg/mL Na_3-citrate	60-100 nm	6 min	1.59E+04
Pym-mc	Batch synthesis yielding macroscopic Pym				N/A	N/A	N/A	-
ZnS	10 mM $Zn(NO_3)_2$	10 mM Na_2S	DI water	DI water	None	40-300	12 min	2.26E+04
Chalcopyrite ($CuFeS_2$)	Batch synthesis indicated in SI.				None	N/A	24 min	4.61E+04
Covellite (CuS)	Milled from naturally grown mineral				None	N/A	24 min	7.14E+04
Chalcocite (Cu_2S)	Milled from naturally grown mineral				None	N/A	24 min	1.13E+04
Pyrite (FeS_2)	Milled from naturally grown mineral				None	N/A	24 min	6.37E+04
Untreated soil	N/A				CMC	N/A	24 min	1.60E+04
P treated Soil	N/A				CMC	N/A	42 min	8.65E+04
Urban lake sediment	N/A				CMC	N/A	30 min	5.55E+04
Marine offshore sediment	N/A				CMC	N/A	66 min	1.38E+05
NGP	N/A				None	N/A	54 min	6.22E+04
Hydraulic fracturing wastewater	N/A				CMC	N/A	24 min	2.09E+04

Table S5. Results of shell-by-shell fitting to the Hg L_{III} EXAFS spectra of sample NGP and the model compound *β*-HgS (metacinnabar) for comparison. The amplitude reduction factor (S_0^2) was simultaneously fitted to both datasets. For *β*-HgS, the number of degenerate S atoms (or coordination number (CN)) was fixed to the literature value of 4 [22] to obtain an independent value of S_0^2. ΔE represents the energy shift between the calculated path and the measured spectra. σ_2 represents the mean square variation in the path length combining thermal and static disorder. The numbers in parentheses indicate the uncertainty in the last digit. Results are plotted in Figure S8cd.

Sample	S_0^2	CN	*R* (Å)	Δ*E* (eV)	σ^2 (Å^2)
β-HgS	0.74(5)	4	2.545(6)	6.6(7)	0.0073(8)
NGP		3.3(7)	2.55(4)	7.4(5)	0.007(6)
Literature values for *β*-HgS					
	-	4	2.534	-	-

S12. Supporting Figures

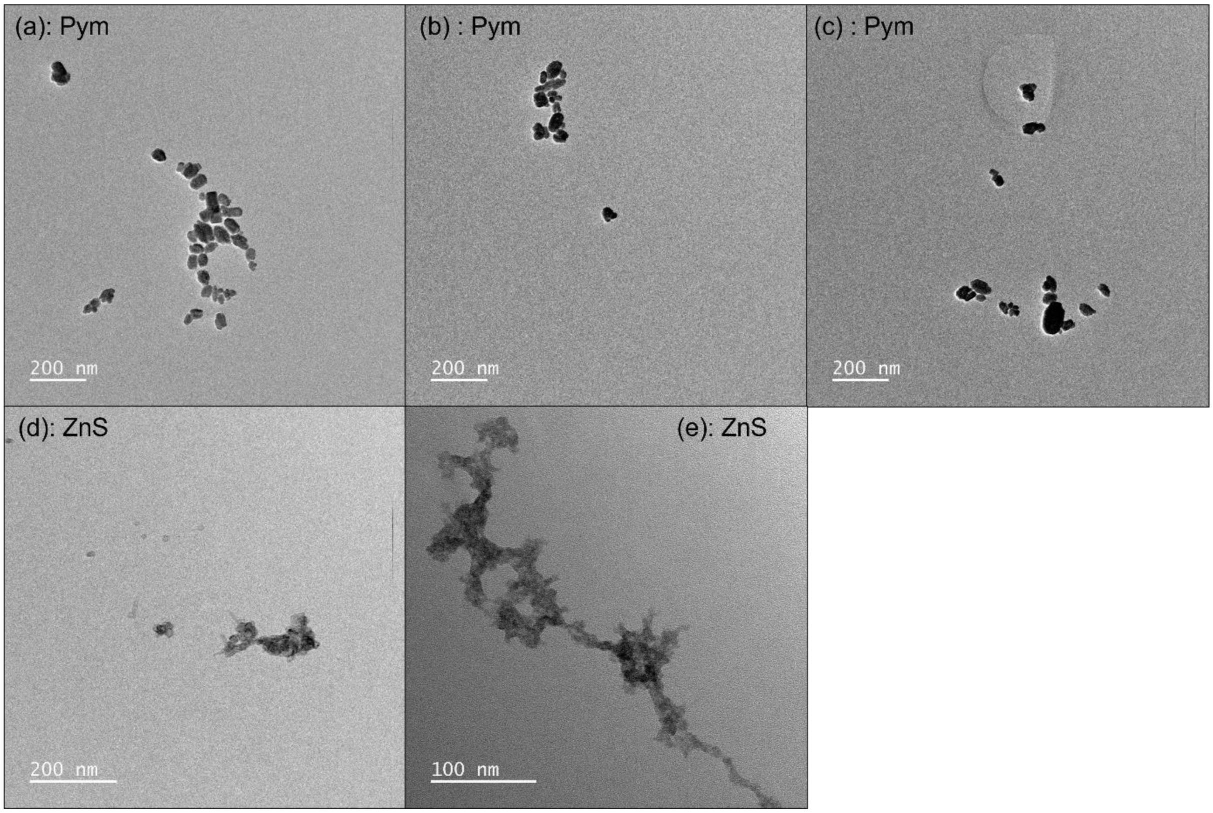

Figure S1. Transmission electron microscopy (TEM) micrographs recorded on samples Pym (a-c) and ZnS (d-e).

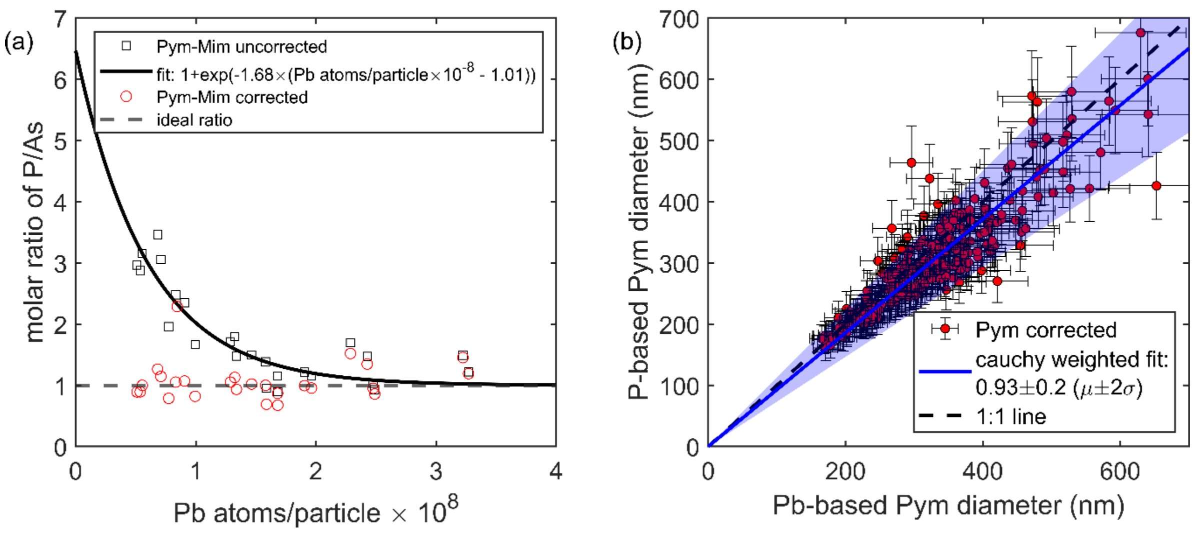

Figure S2. Samples Pym and Pym-Mim. (a) Ratio of P/As in particle events as a function of Pb (atoms/particle), representing the particle size of sample Pym-Mim. The open, black squares represent the original data, the bold, black line represents an exponential decay fitted (correction function) to the data, the open, red circles present the corrected data (original data / correction function), and the dashed, gray line represents the ideal ratio of unity applied during Pym-Mim preparation. (b) Data recorded on sample Pym (same as displayed in Figure 1a), corrected using the correction function shown in (a). This graph can be compared to Figure 1a.

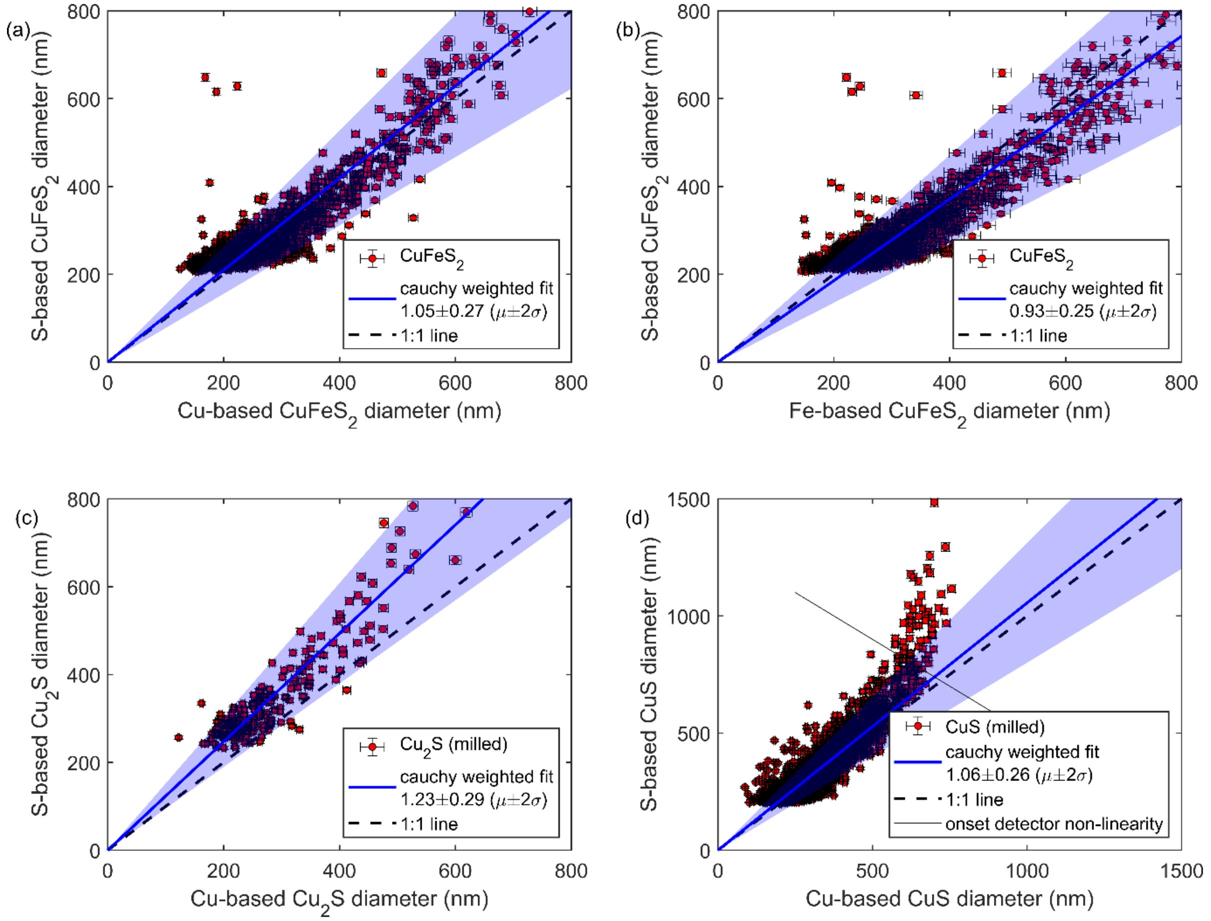

Figure S3. (a) Sp-icpTOF-MS data recorded on $CuFeS_2$. The $CuFeS_2$ (ρ_{CuFeS2} = 4.2 g/cm^3) diameter calculated from the S-signal was plotted *versus* the $CuFeS_2$ diameter calculated from the Cu-signal. An ideal measurement would fall on the dashed 1 to 1 line. Error bars indicate two standard deviations propagated from the uncertainty in the calibrations. The solid, blue line indicate the results of a cauchy weighted zero-forced intercept fit to the data. The blue shaded areas indicate ±2σ of the fitting results. This applies to all panels in this figure. (b) Sp-icpTOF-MS data recorded on $CuFeS_2$ (same data as in (a)). The $CuFeS_2$ diameter calculated from the S-signal was plotted *versus* the $CuFeS_2$ diameter calculated from the Fe-signal. (c) Sp-icpTOF-MS data recorded on Cu_2S (ρ_{Cu2S} = 5.65 g/cm^3). The Cu_2S diameter calculated from the S-signal was plotted *versus* the Cu_2S diameter calculated from the Cu-signal. (d) Sp-icpTOF-MS data recorded on CuS (ρ_{CuS} = 4.7 g/cm^3). The CuS diameter calculated from the S-signal was plotted *versus* the CuS diameter calculated from the Cu-signal. Cu_2S and CuS (milled) were obtained by milling minerals separated from a host rock, whereas $CuFeS_2$ was synthesized.

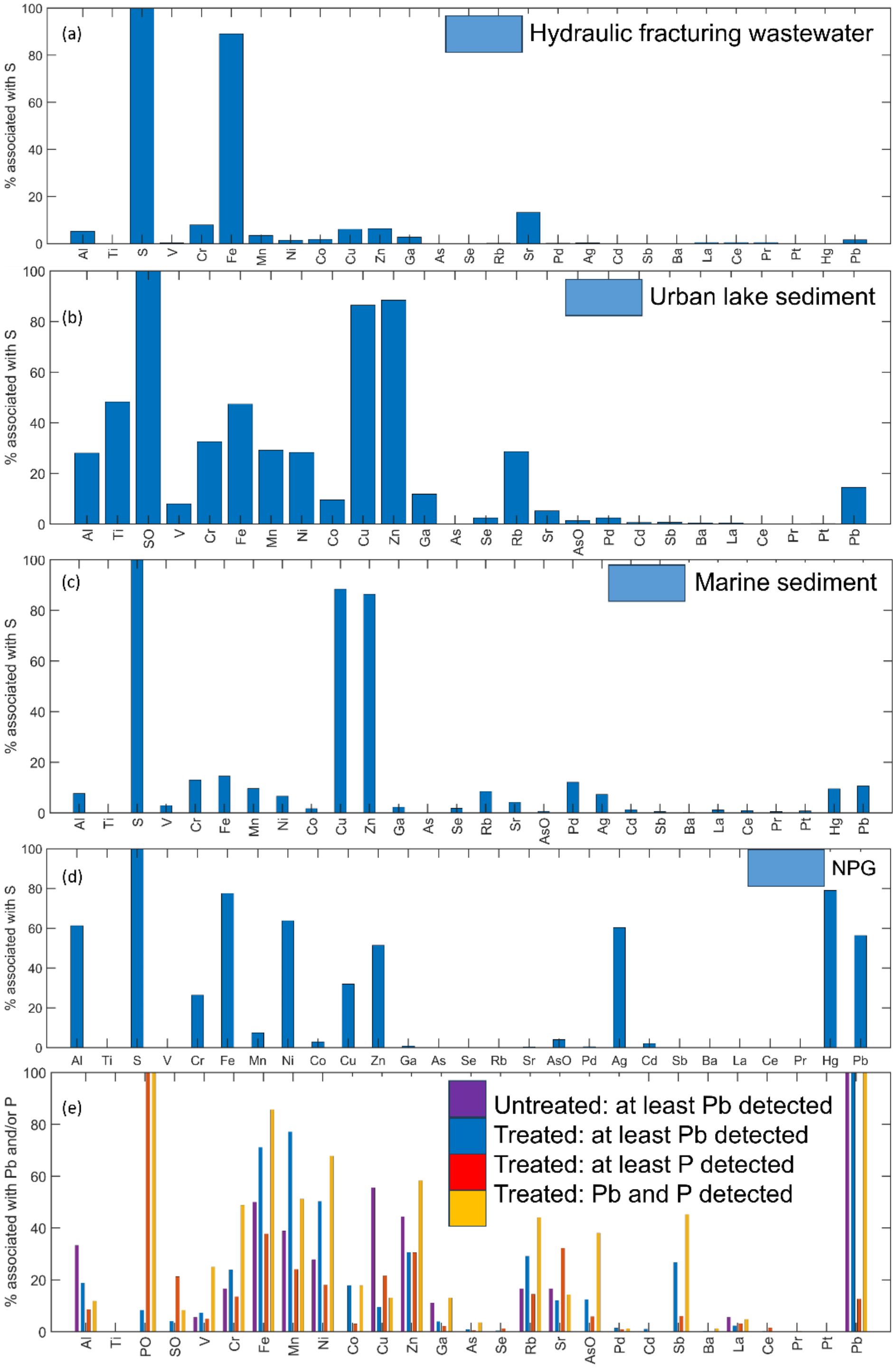

Figure S4. Shows what percentage of particles (by number concentration) is associated with sulfur (a-d) or with P and/or Pb (e). Sample: (a) urban lake sediment; (b) hydraulic fracturing wastewater; (c) marine sediment; (d) NGP from an industrial process stream; (e) untreated and treated soil. Bar chart of associated elements with particles in which Pb and P (blue bars: treated soil), at least Pb (violet bars: untreated soil, red bars: treated soil) and at least P (yellow bars: treated soil) were detected. Values are given in percent (%). For example, in sample NGP, about 80% of the detected particles containing S also contain Hg.

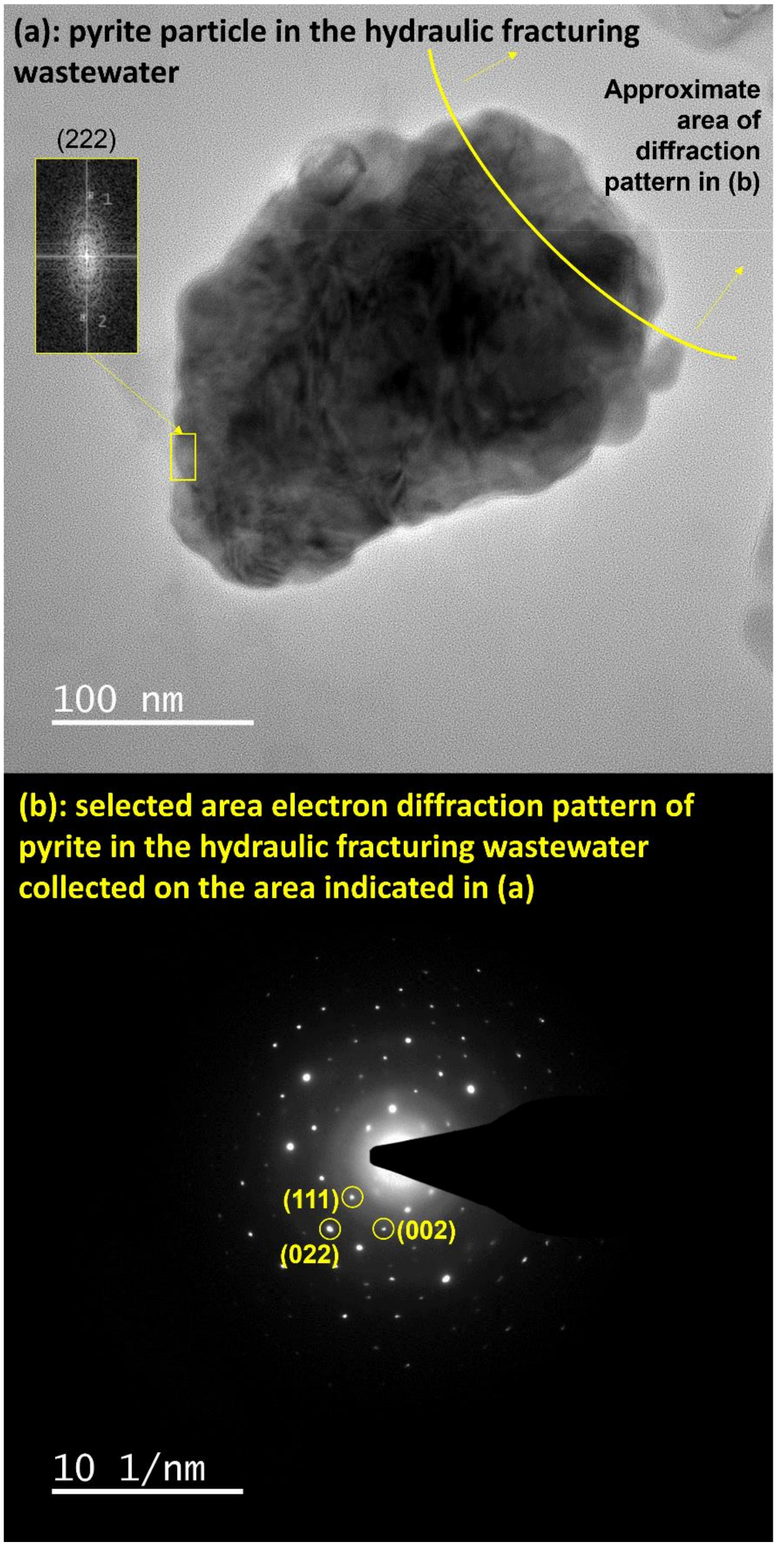

Figure S5. Sample hydraulic fracturing wastewater. (a) Electron micrograph of a ~300 nm colloid containing multiple pyrite crystallinities. A d-spacing obtained from a Fast Fourier Transform (FFT) (inset) showed

good agreement to a d-spacing for pyrite obtained from the literature.[23] (b) Selected area electron diffractogram of multiple pyrite crystallites recorded on the approximate area indicated in panel (a).

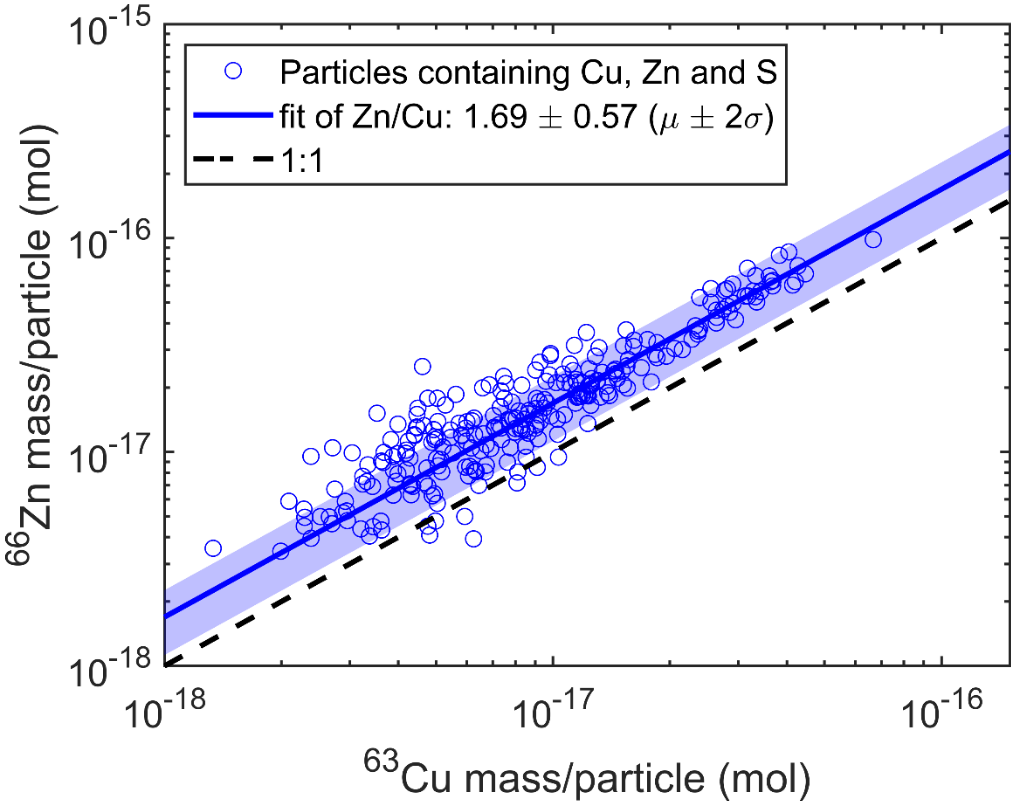

Figure S6. Sp-icpTOF-MS data recorded on the subaquatic urban lake sediment sample. Displayed are all particle events in which Cu, Zn and S were detected in moles/particle. Zn (mol/particle) (y-axis) *versus* Cu (mol/particle) (x-axis).

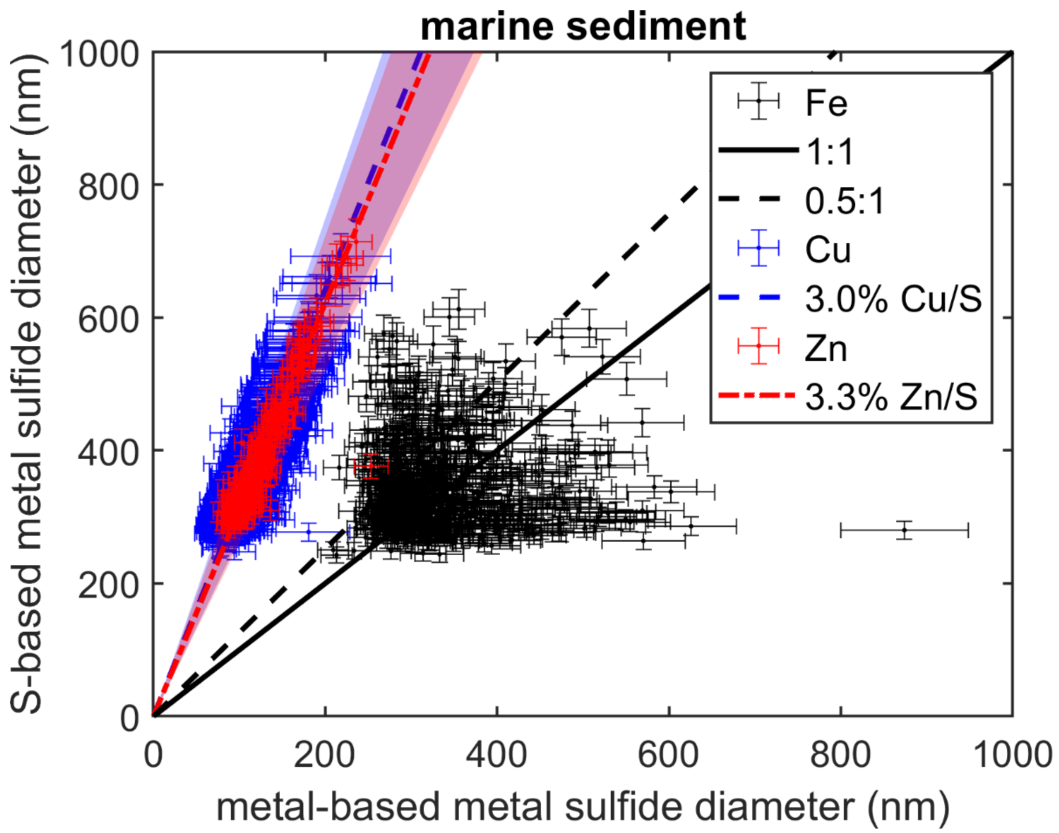

Figure S7. Sp-icpTOF-MS data recorded on the marine sediment sample. S-based metal sulfide diameter versus metal based metal sulfide diameter for FeS (black dots), CuS (blue dots) and ZnS (red dots). Error bars represent ±2σ calculated from the uncertainty in the calibration curves. The dashed, blue line indicates 3.0mol% Cu replacing Fe in FeS and the dashed-dotted, red line 3.3mol% Zn replacing Fe in FeS. Both values represent fitting results, and the shaded areas indicate ±2σ. Only every 20th data point of the Zn data (5%) is shown for clarity. The regression and confidence interval were calculated on the full dataset.

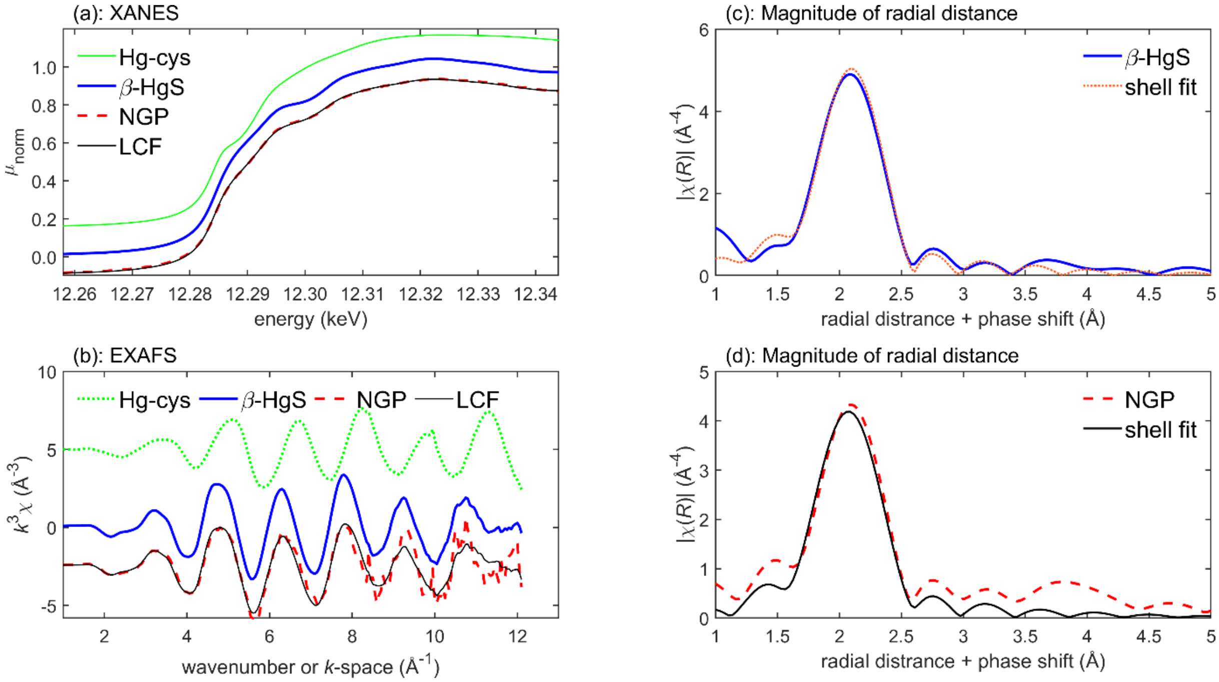

Figure S8. XAS results collected on sample NGP, the model compounds metacinnabar (β-HgS) and Hg bound to cysteine (Hg-cys) and linear combination fitting (LCF) results to NGP using β-HgS and Hg-cys. (a) X-ray absorption near edge structure (XANES) spectra. (b) Extended X-ray absorption fine structure spectra. The dotted, green lines indicate Hg-cys, the solid, blue lines indicate β-HgS, the dashed, red lines indicate NGP, and the thin black lines indicate the LCF results. (c) Shows magnitude of the Fast Fourier Transform (FFT) of the β-HgS EXAFS (solid, blue line) and the result of a shell fitting to the data (dotted, orange line). (d) Shows magnitude of the FFT of NGP EXAFS (dashed, red line) and the result of a shell fit to the data (solid, black line). Results of the shell fittings are tabulated (Table S5).

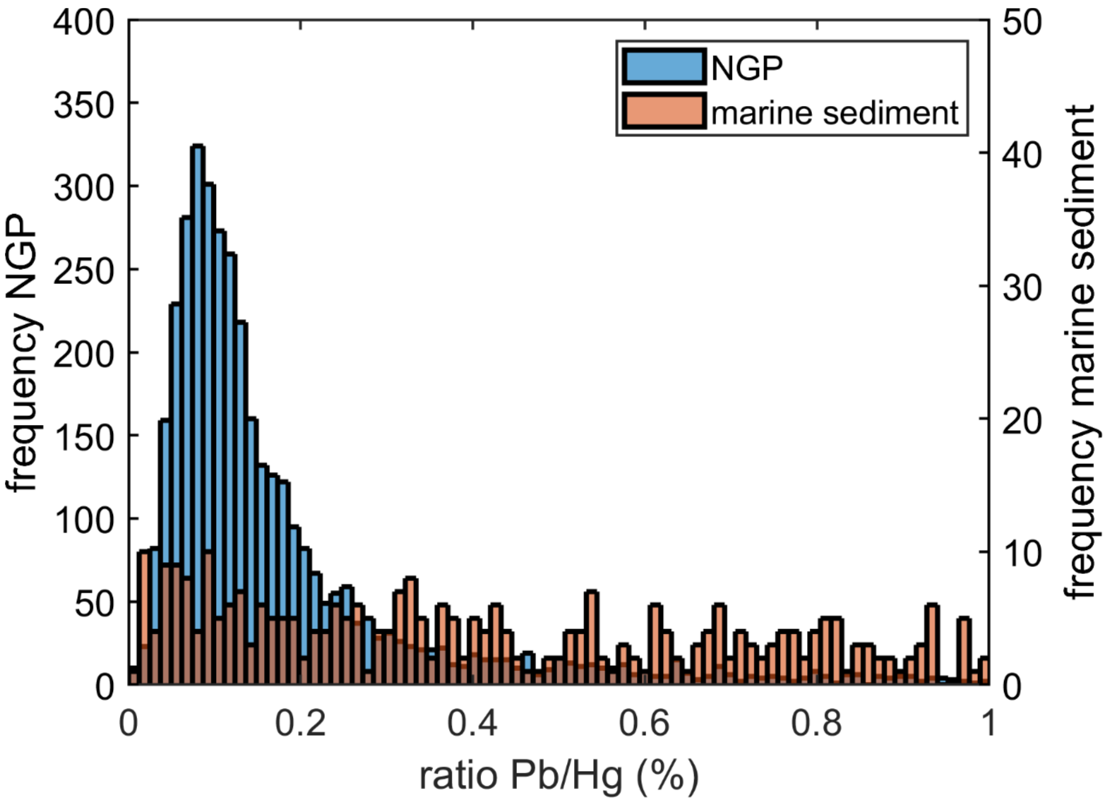

Figure S9. Samples NGP and marine sediment. Histogram of the ratio of Pb to Hg (in wt%) for samples NGP on the left axis and for the sample marine sediment on the right axis. Comparable data is shown for Ag/Hg (in wt%) in Figure 3d.

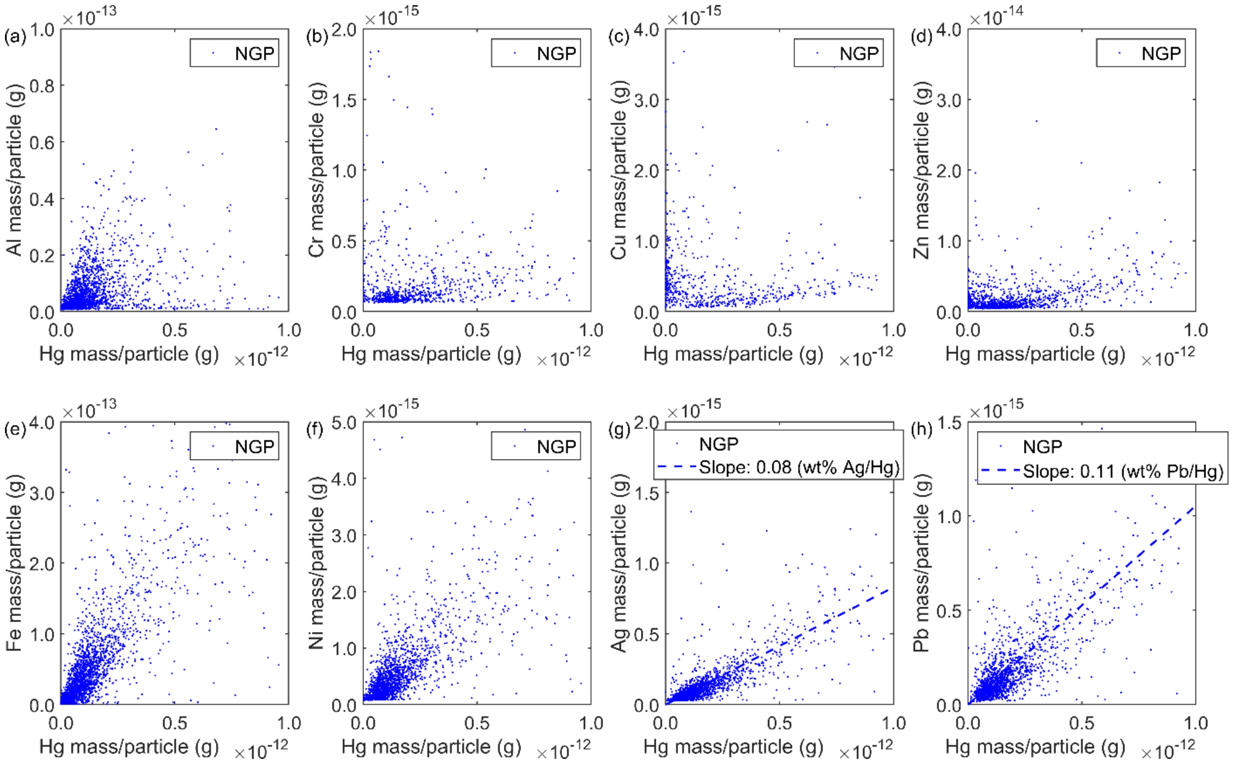

Figure S10. Sample NGP. Scatter plots of As, Cr, Cu, Zn, Fe, Ni, Ag or Pb *versus* Hg. Regression results are plotted for Ag *versus* Hg and Pb *versus* Hg. The purpose of this was to demonstrate that only some of the masses of the frequently co-occurring elements in Figure S4 were correlated to Hg. Histograms of these data (Ag/Hg and Pb/Hg) are displayed in Figure 3d and Figure S9.

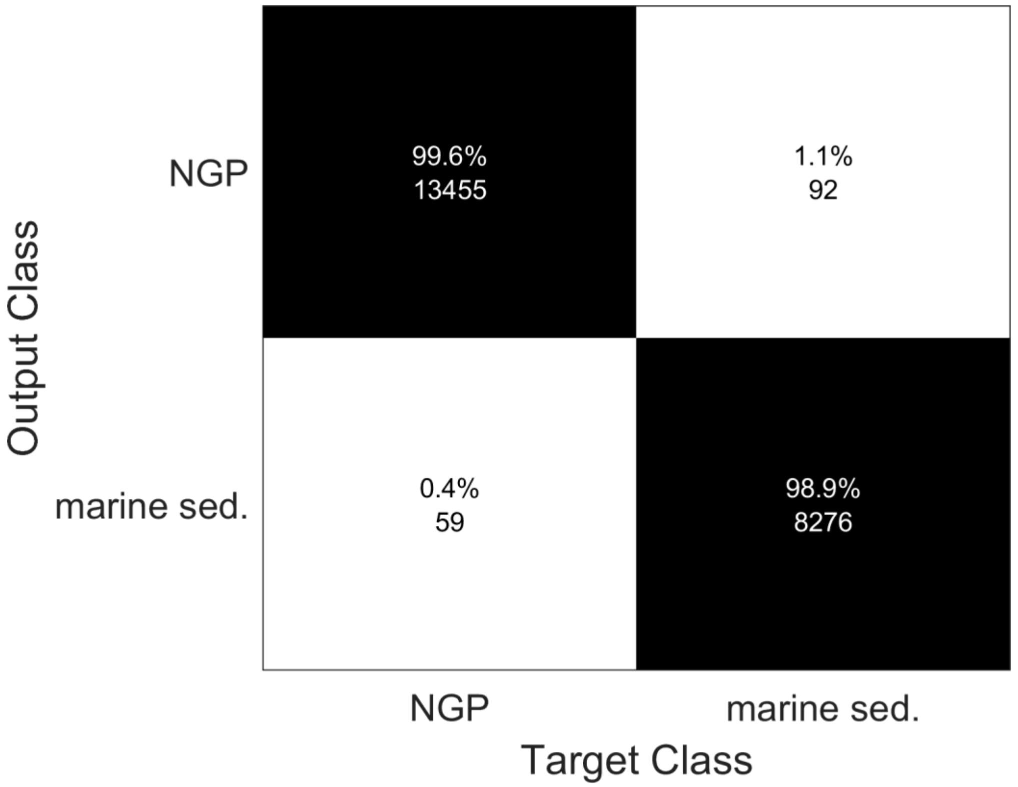

Figure S11. Confusion matrix of k means clustering results applied on the marine sediment and NGP sample. Details of the computation are described in SI section S9.

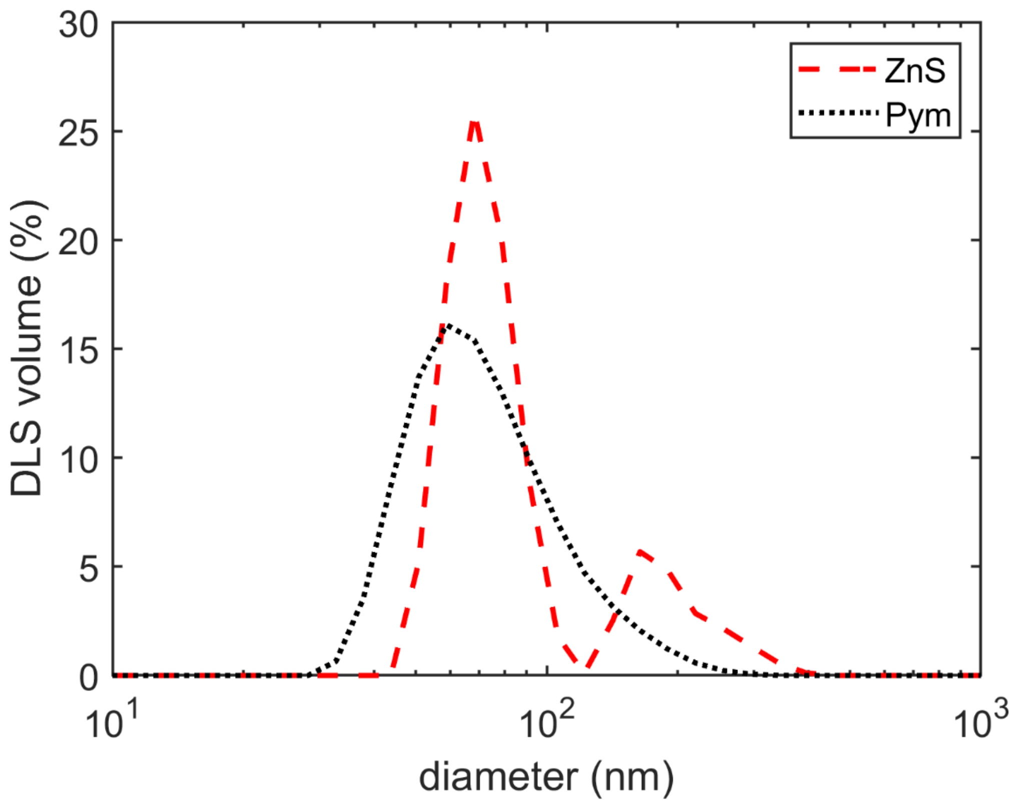

Figure S12. Dynamic light scattering (DLS) results of the synthesized zinc sulfide (ZnS) and pyromorphite (Pym). The data of sample Pym is also displayed in Figure S21 for comparison with sp-icpTOF-MS data.

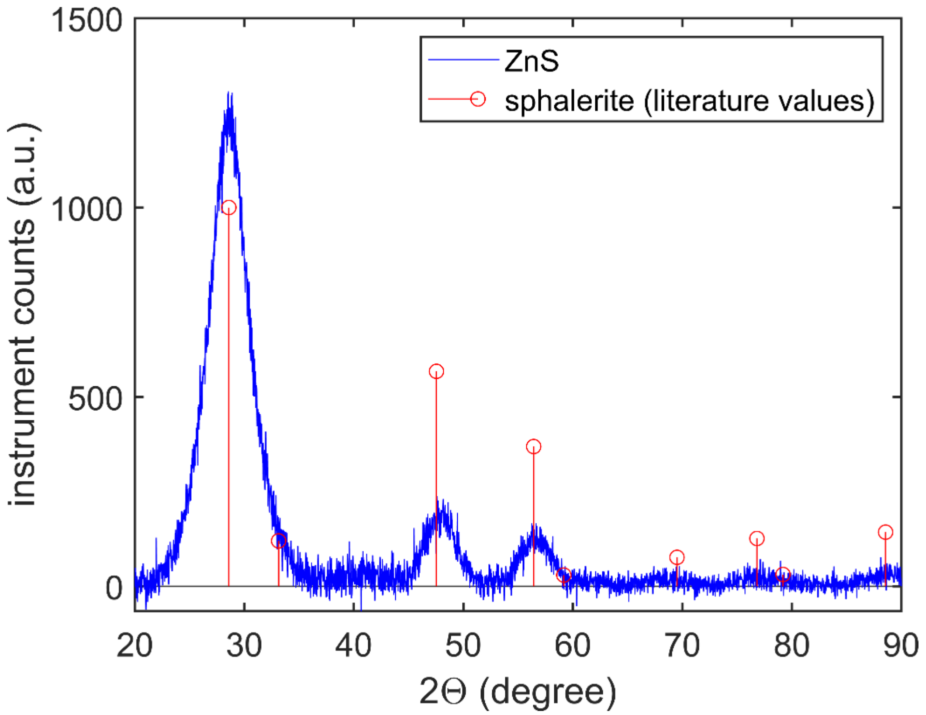

Figure S13. X-ray powder diffraction spectrum of flash-precipitated ZnS and literature values for sphalerite [24].

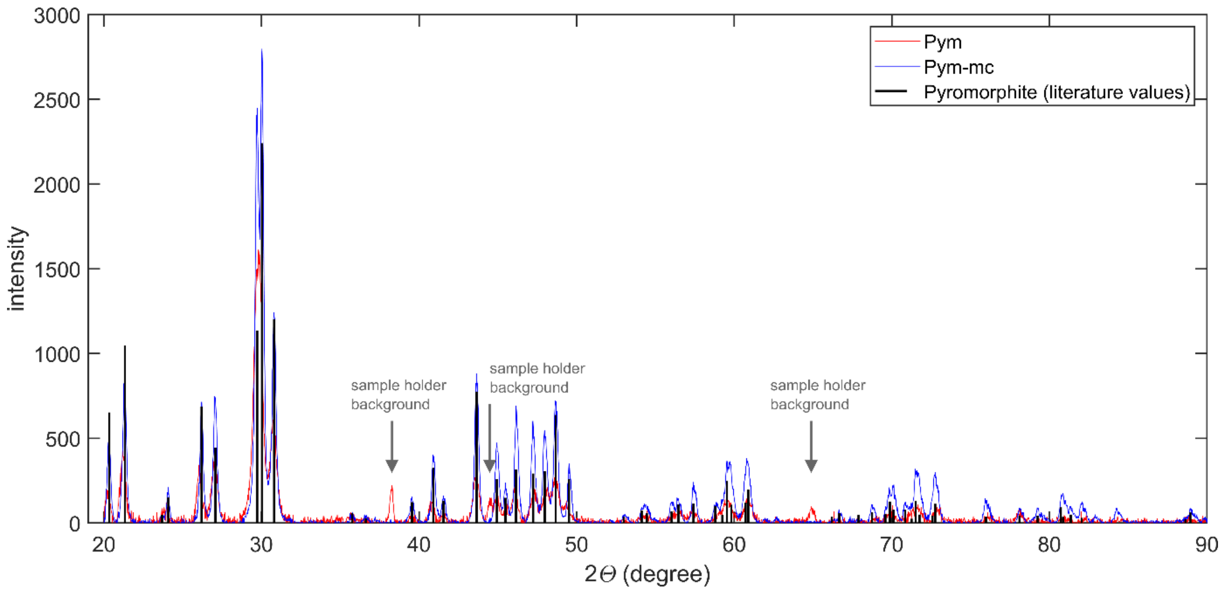

Figure S14. X-ray diffraction data recorded on flash-precipitated Pym (red curve) and batch synthesized Pym-mc (blue curve). Vertical, black bars indicate a diffraction pattern obtained from the literature. [25]

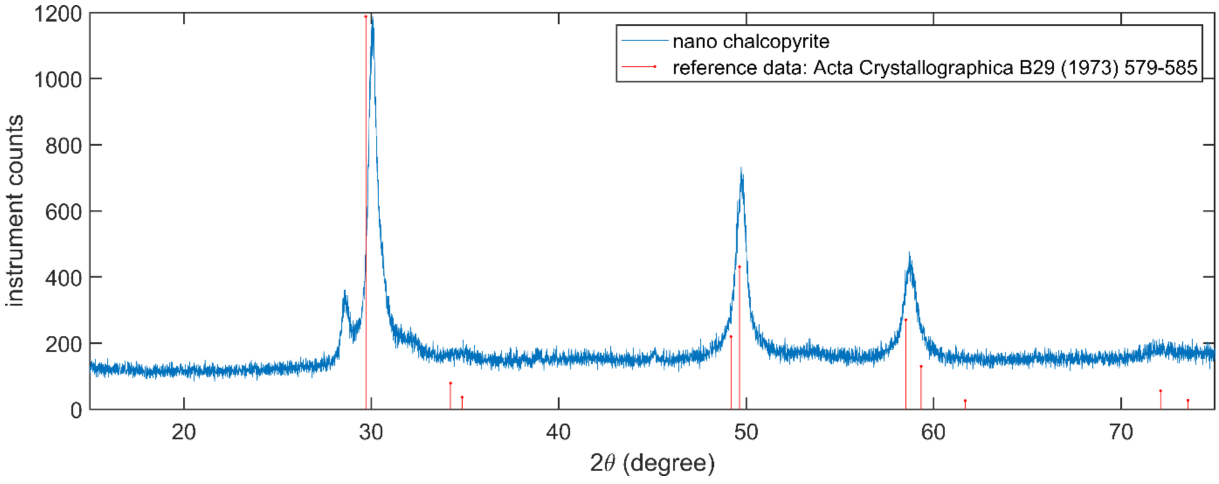

Figure S15. X-ray diffraction data recorded on the nano-chalcopyrite ($CuFeS_2$) sample. Reference data obtained from the literature.[26]

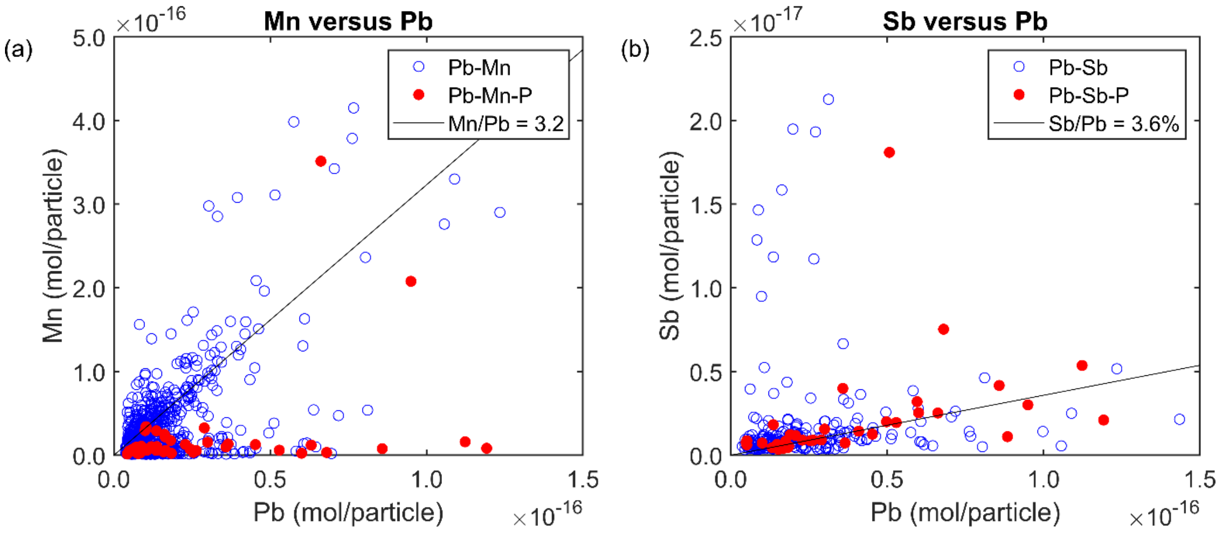

Figure S16. P treated contaminated soil sample data. (a) Mn (mol/particle) *versus* Pb (mol/particle). Open, blue circles indicate particles that did not contain P, whereas filled, red circles indicate particles that also contained P. (b) Shows the same data but for Sb *versus* Pb. Lines in indicate the ratio of Mn to Pb of 3.2 and Sb to Pb of 3.6mol/mol%, respectively.

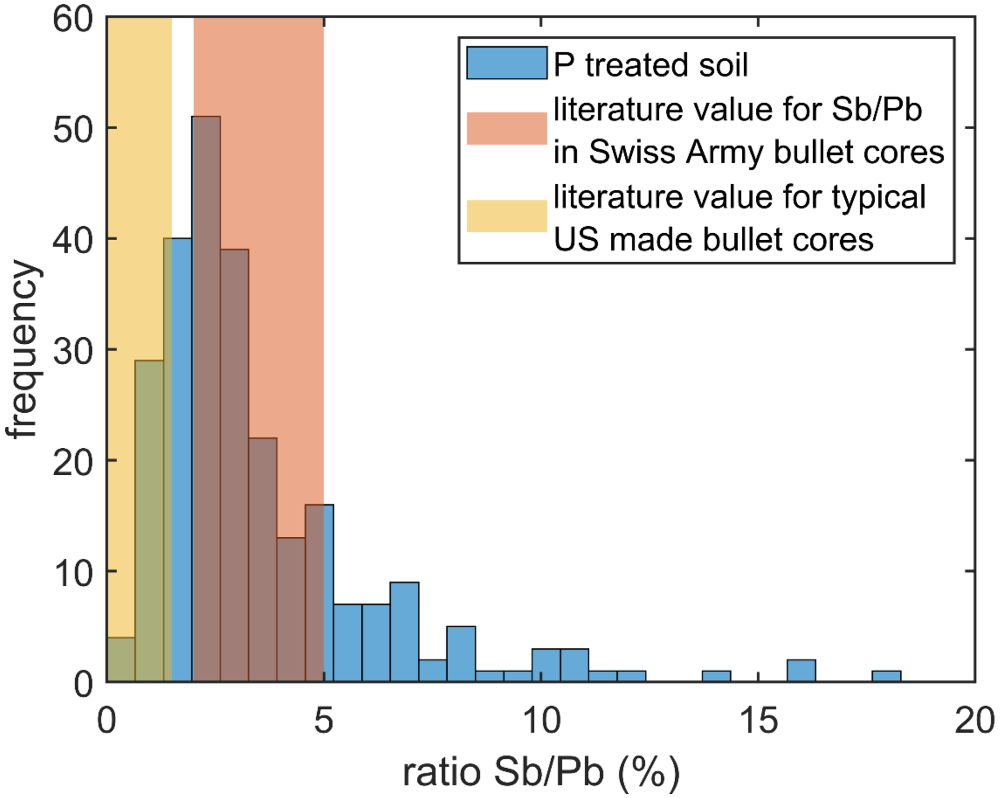

Figure S17. P treated contaminated soil sample. Histogram of the frequency of the ratio of Sb/Pb (w/w%). The red and yellow shaded areas indicate ratios found in Swiss Army bullets cores [19] and bullets produced by a large US ammunition manufacturer.[27]

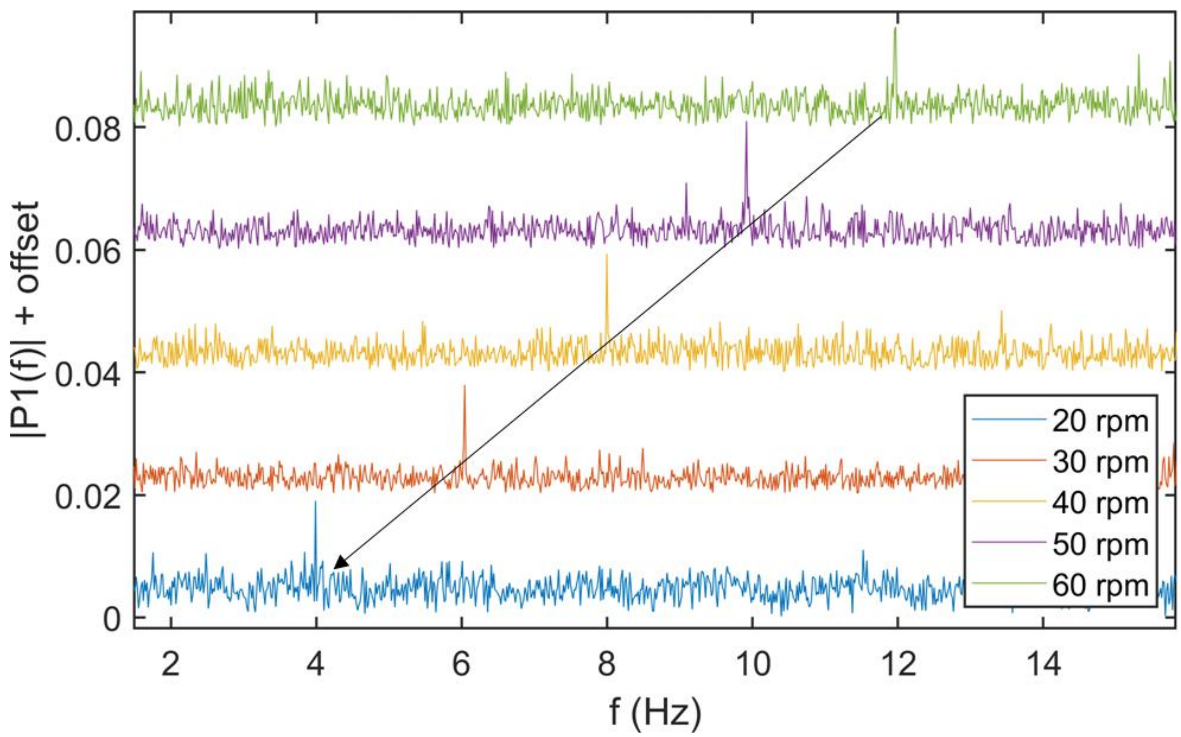

Figure S18. Single sided amplitude spectrum (|P1(f)|) as a function of frequency (Hz). The measured sample contained 100 ppt Au-NP (*d* = 40 nm NanoXact, Nanocomposix, US) and 2 ppb of a dissolved Au background (ICP standard solution, Merck) in DI water. The peristaltic pump rotation speed varied between 20 and 60 rpm.

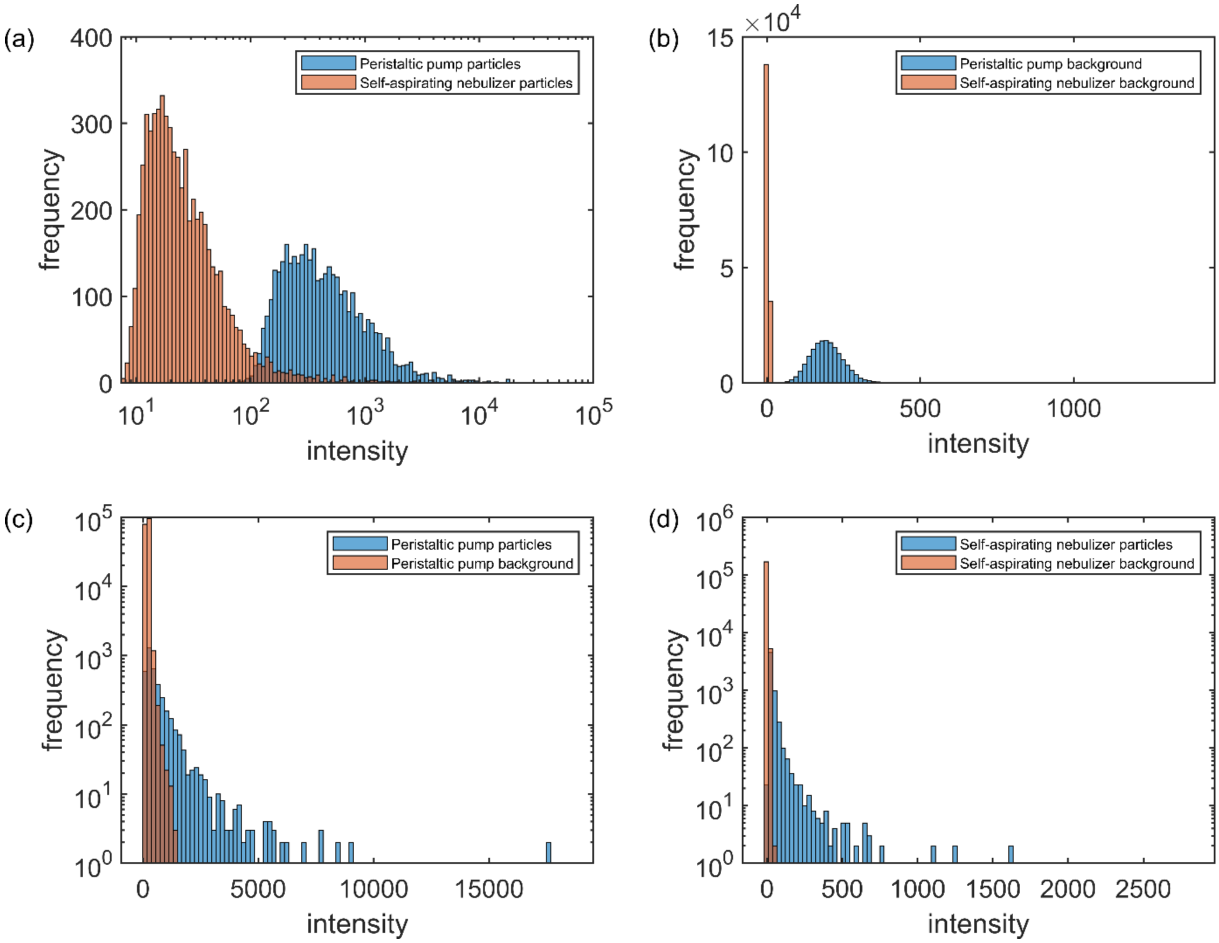

Figure S19. Sample Pym, all signals are ^{208}Pb. (a) Particle events collected using the peristaltic pump sample introduction (blue, n = 3841) and a self-aspirating nebulizer (red, n = 6133). (b) Background signal collected using the peristaltic pump sample introduction and a self-aspirating nebulizer. (c) For comparison, the particle events (blue) and background (red) collected using the peristaltic pump (data from (a) and (b)). (d) For comparison, the particle events (blue) and background (red) collected a self-aspirating nebulizer (data from (a) and (b)).

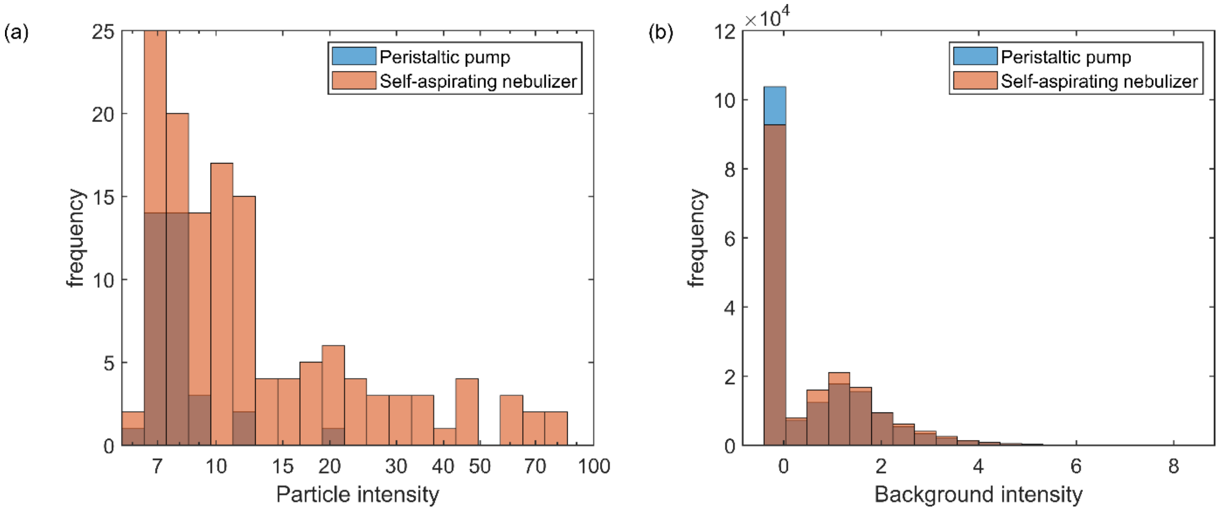

Figure S20. Sample Pym, all signals are $^{31}P^{16}O$. (a) Particle events collected using the peristaltic pump sample introduction (blue, *n* = 35) and a self-aspirating nebulizer (red, *n* = 137). (b) Background signal collected using the peristaltic pump sample introduction and a self-aspirating nebulizer.

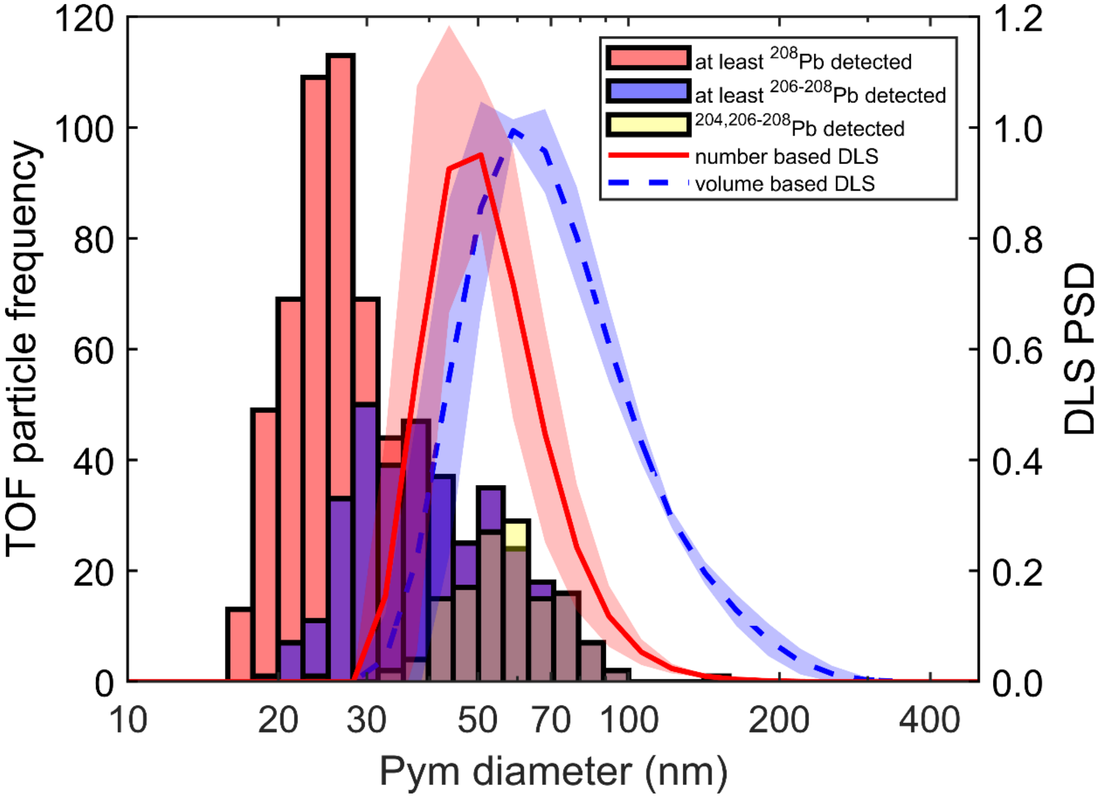

Figure S21. Sample Pym. Sp-icpTOF-MS detected particle frequency (left y-axis) and dynamic light scattering (DLS) particle size distribution (PSD) (right y-axis) *versus* the Pym diameter (nm). Differently colored bars indicate the number of isotopes included in the analysis. The bold, red line and the dashed, blue

line indicate the number and volume based DLS PSD, respectively. The shaded areas indicate uncertainty (±2σ) after recording a total of 60 spectra.

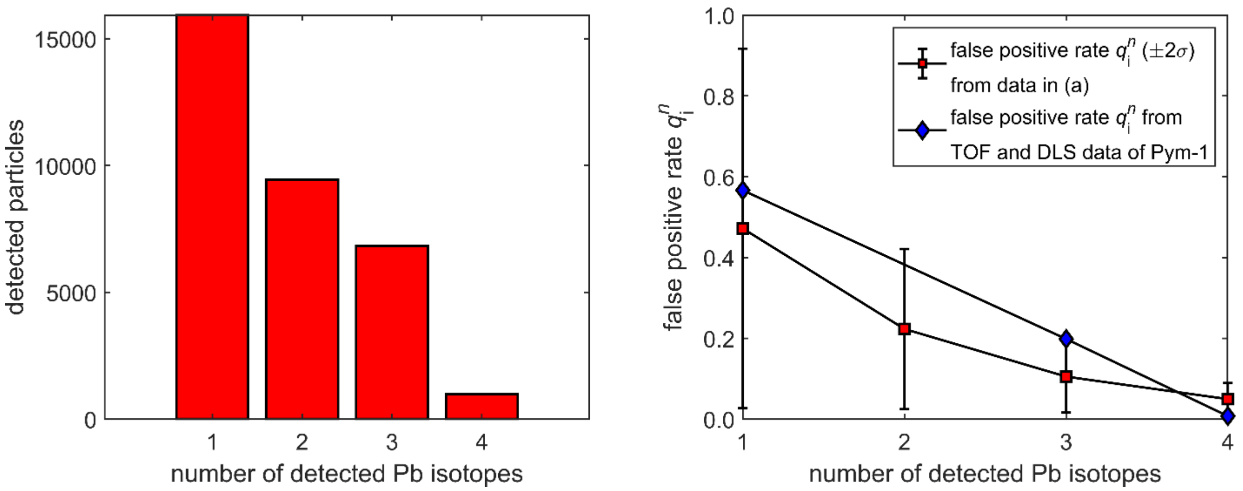

Figure S22. (a) Bar chart of the number of particles detected with at least 1-4 Pb isotopes present. This number is equivalent to the variable *p* in eq. S1-S4. (b) The calculated false positive rate (red squares) as a function of the number of Pb isotopes included in the analysis. Blue diamonds represent the false positive rate determined by the share of all Pym particles determined by sp-icpTOF-MS that are smaller than the lower end of the DLS determined size distribution, *i.e.*, 30 nm (Figure S21).

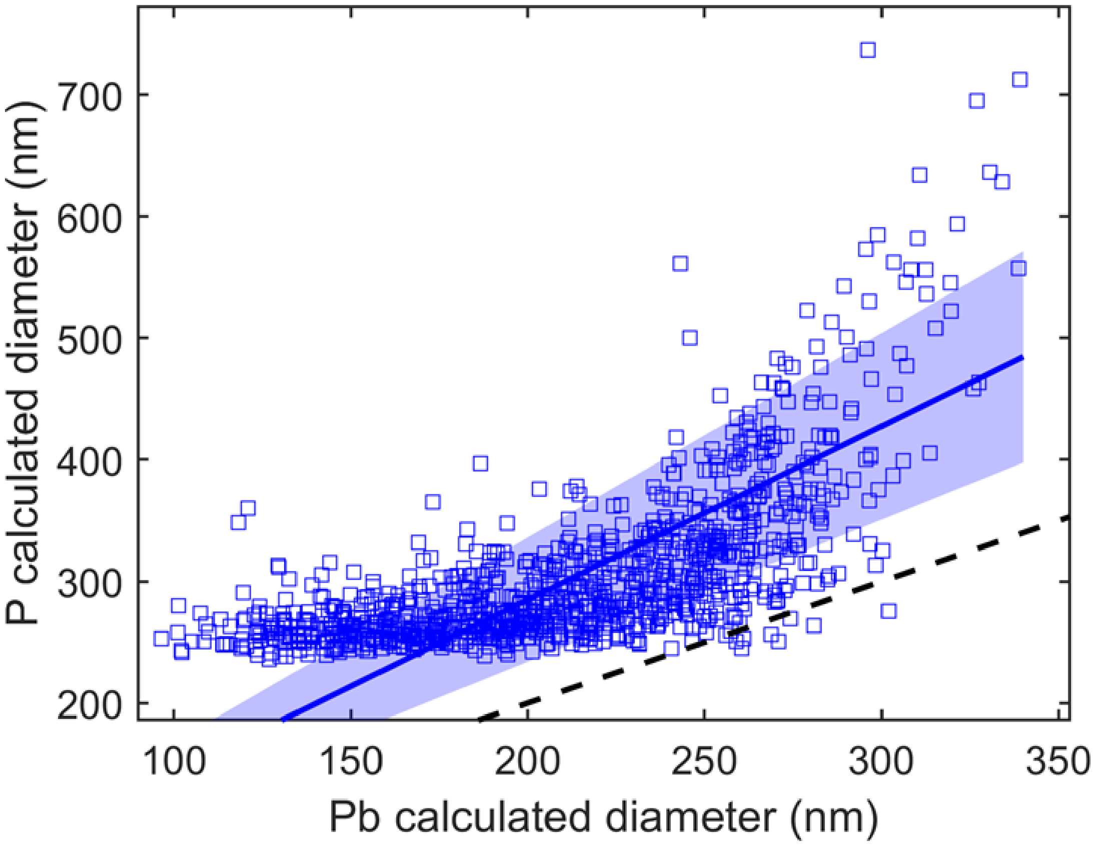

Figure S23. Sample Pym measured without a notch filter set to m/z 209 (close to the mass to charge ratios of Pb at 204 to 208) as discussed in section S3. The dashed, black line indicates where an ideal measurement should fall, the solid, blue line indicates fitting results and the shaded areas indicate ±2σ of the fit.

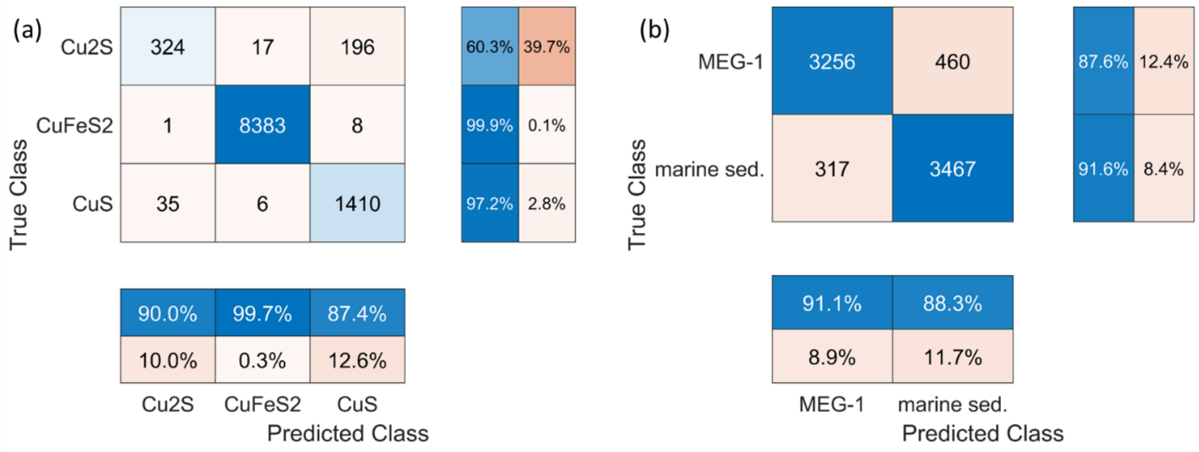

Figure S24. Confusion matrices obtained from applying logistic regression to distinguishing (a) CuS, Cu_2S and $CuFeS_2$ or (b) pure HgS in a marine sediment from impure HgS in a water-laden mono ethylene glycol sampled from an industrial process. These graphs can be compared to the confusion matrices in Figure 1f and Figure S11 in which results from applying k means clustering are displayed.

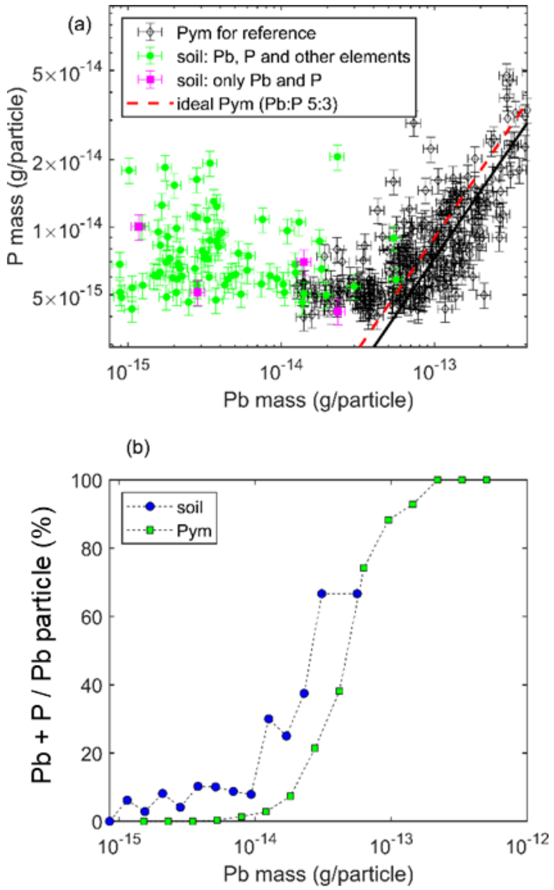

Figure S25. P-treated soil and Pym for reference. (a) P *versus* Pb (both g/particle) for Pym (open, black diamonds), and the soil data. Green circles indicate particles that contained Pb, P and other isotopes, whereas magenta squares indicate particles where only Pb and P were detected. Approximate Pym-size equivalents in the soil data calculated from Pb mass would range from 70 to 250 nm. (b) Relative occurrence of Pb-P particles to Pb particles are indicated for soil (blue circles) and Pym (green squares) in percentage.

S13. References

(1) Tanner, M.; Günther, D. Short Transient Signals, a Challenge for Inductively Coupled Plasma Mass Spectrometry, a Review. *Analytica Chimica Acta* **2009**, *633* (1), 19–28. https://doi.org/10.1016/j.aca.2008.11.041.
(2) Hendriks, L.; Gundlach-Graham, A.; Günther, D. Performance of Sp-ICP-TOFMS with Signal Distributions Fitted to a Compound Poisson Model. *Journal of Analytical Atomic Spectrometry* **2019**, *34* (9), 1900–1909. https://doi.org/10.1039/C9JA00186G.
(3) Lee, S.; Bi, X.; Reed, R. B.; Ranville, J. F.; Herckes, P.; Westerhoff, P. Nanoparticle Size Detection Limits by Single Particle ICP-MS for 40 Elements. *Environmental Science & Technology* **2014**, *48* (17), 10291–10300. https://doi.org/10.1021/es502422v.

(4) Pace, H. E.; Rogers, N. J.; Jarolimek, C.; Coleman, V. A.; Higgins, C. P.; Ranville, J. F. Determining Transport Efficiency for the Purpose of Counting and Sizing Nanoparticles via Single Particle Inductively Coupled Plasma Mass Spectrometry. *Analytical Chemistry* **2011**, *83* (24), 9361–9369. https://doi.org/10.1021/ac201952t.
(5) Liu, Y.; Cheng, C.; Liu, Y.; Prud'homme, R. K.; Fox, R. O. Mixing in a Multi-Inlet Vortex Mixer (MIVM) for Flash Nano-Precipitation. *Chemical Engineering Science* **2008**, *63* (11), 2829–2842. https://doi.org/10.1016/j.ces.2007.10.020.
(6) Markwalter, C. E.; Pagels, R. F.; Wilson, B. K.; Ristroph, K. D.; Prud'homme, R. K. Flash NanoPrecipitation for the Encapsulation of Hydrophobic and Hydrophilic Compounds in Polymeric Nanoparticles. *JoVE* **2019**, No. 143, e58757. https://doi.org/10.3791/58757.
(7) Wang, X.; Huang, H.; Liang, B.; Liu, Z.; Chen, D.; Shen, G. ZnS Nanostructures: Synthesis, Properties, and Applications. *Critical Reviews in Solid State and Materials Sciences* **2013**, *38* (1), 57–90. https://doi.org/10.1080/10408436.2012.736887.
(8) Topolska, J.; Manecki, M.; Bajda, T.; Borkiewicz, O.; Budzewski, P. Solubility of Pyromorphite Pb5(PO4)3Cl at 5–65°C and Its Experimentally Determined Thermodynamic Parameters. *The Journal of Chemical Thermodynamics* **2016**, *98*, 282–287. https://doi.org/10.1016/j.jct.2016.03.031.
(9) Baker, L. R.; Pierzynski, G. M.; Hettiarachchi, G. M.; Scheckel, K. G.; Newville, M. Micro-X-Ray Fluorescence, Micro-X-Ray Absorption Spectroscopy, and Micro-X-Ray Diffraction Investigation of Lead Speciation after the Addition of Different Phosphorus Amendments to a Smelter-Contaminated Soil. *Journal of Environmental Quality* **2014**, *43* (2), 488–497. https://doi.org/10.2134/jeq2013.07.0281.
(10) Baldi, F.; Parati, F.; Filippelli, M. Dimethylmercury and Dimethylmercury-Sulfide of Microbial Origin in the Biogeochemical Cycle of HG. *Water, Air, and Soil Pollution* **1995**, *80* (1), 805–815. https://doi.org/10.1007/BF01189732.
(11) Vantelon, D.; Lanzirotti, A.; Scheinost, A. C.; Kretzschmar, R. Spatial Distribution and Speciation of Lead around Corroding Bullets in a Shooting Range Soil Studied by Micro-X-Ray Fluorescence and Absorption Spectroscopy. *Environmental Science & Technology* **2005**, *39* (13), 4808–4815. https://doi.org/10.1021/es0482740.
(12) Hashimoto, Y.; Matsufuru, H.; Takaoka, M.; Tanida, H.; Sato, T. Impacts of Chemical Amendment and Plant Growth on Lead Speciation and Enzyme Activities in a Shooting Range Soil: An X-Ray Absorption Fine Structure Investigation. *Journal of Environmental Quality* **2009**, *38* (4), 1420–1428. https://doi.org/10.2134/jeq2008.0427.
(13) Munksgaard, N. C.; Lottermoser, B. G. Fertilizer Amendment of Mining-Impacted Soils from Broken Hill, Australia: Fixation or Release of Contaminants? *Water, Air, & Soil Pollution* **2011**, *215* (1), 373–397. https://doi.org/10.1007/s11270-010-0485-y.
(14) Montaño, M. D.; Cuss, C. W.; Holliday, H. M.; Javed, M. B.; Shotyk, W.; Sobocinski, K. L.; Hofmann, T.; Kammer, F. von der; Ranville, J. F. Exploring Nanogeochemical Environments: New Insights from Single Particle ICP-TOFMS and AF4-ICPMS. *ACS Earth and Space Chemistry* **2022**, *6* (4), 943–952. https://doi.org/10.1021/acsearthspacechem.1c00350.
(15) Della Puppa, L.; Komárek, M.; Bordas, F.; Bollinger, J.-C.; Joussein, E. Adsorption of Copper, Cadmium, Lead and Zinc onto a Synthetic Manganese Oxide. *Journal of Colloid and Interface Science* **2013**, *399*, 99–106. https://doi.org/10.1016/j.jcis.2013.02.029.
(16) Cao, R. X.; Ma, L. Q.; Chen, M.; Singh, S. P.; Harris, W. G. Phosphate-Induced Metal Immobilization in a Contaminated Site. *Environmental Pollution* **2003**, *122* (1), 19–28. https://doi.org/10.1016/S0269-7491(02)00283-X.
(17) Scheckel, K. G.; Ryan, J. A. Spectroscopic Speciation and Quantification of Lead in Phosphate-Amended Soils. *Journal of Environmental Quality* **2004**, *33* (4), 1288–1295. https://doi.org/10.2134/jeq2004.1288.

(18) Rhee, Y. J.; Hillier, S.; Gadd, G. M. Lead Transformation to Pyromorphite by Fungi. *Current Biology* **2012**, *22* (3), 237–241. https://doi.org/10.1016/j.cub.2011.12.017.
(19) Scheinost, A. C.; Rossberg, A.; Vantelon, D.; Xifra, I.; Kretzschmar, R.; Leuz, A.-K.; Funke, H.; Johnson, C. A. Quantitative Antimony Speciation in Shooting-Range Soils by EXAFS Spectroscopy. *Geochimica et Cosmochimica Acta* **2006**, *70* (13), 3299–3312. https://doi.org/10.1016/j.gca.2006.03.020.
(20) Pedregosa, F.; Varoquaux, G.; Gramfort, A.; Michel, V.; Thirion, B.; Grisel, O.; Blondel, M.; Prettenhofer, P.; Weiss, R.; Dubourg, V. Scikit-Learn: Machine Learning in Python. *the Journal of machine Learning research* **2011**, *12*, 2825–2830.
(21) Bland, G. D.; Battifarano, M.; Liu, Q.; Yang, X.; Lu, D.; Jiang, G.; Lowry, G. V. Single-Particle Metal Fingerprint Analysis and Machine Learning Pipeline for Source Apportionment of Metal-Containing Fine Particles in Air. *Environmental Science & Technology Letters* **2022**. https://doi.org/10.1021/acs.estlett.2c00835.
(22) Zemann, J. Crystal Structures, Vol. 1 by RWG Wyckoff. *Acta Crystallographica* **1965**, *18* (1), 139–139.
(23) Ramsdell, L. S. The Crystal Structure of Some Metallic Sulfides*. *American Mineralogist* **1925**, *10* (9), 281–304.
(24) Skinner, B. J. Unit-Cell Edges of Natural and Synthetic Sphalerites*. *American Mineralogist* **1961**, *46* (11–12), 1399–1411.
(25) Mills, S. J.; Ferraris, G.; Kampf, A. R.; Favreau, G. Twinning in Pyromorphite: The First Documented Occurrence of Twinning by Merohedry in the Apatite Supergroup. *American Mineralogist* **2012**, *97* (2–3), 415–418. https://doi.org/10.2138/am.2012.3984.
(26) Hall, S. R.; Stewart, J. M. The Crystal Structure Refinement of Chalcopyrite, CuFeS2. *Acta Crystallographica Section B*, 1973, *29*, 579–585.
(27) Randich, E.; Duerfeldt, W.; McLendon, W.; Tobin, W. A Metallurgical Review of the Interpretation of Bullet Lead Compositional Analysis. *Forensic Science International* **2002**, *127* (3), 174–191. https://doi.org/10.1016/S0379-0738(02)00118-4.
